# Evaluating Neural Networks Architectures for Competency Prediction from Process Data Using PISA Computer-Based Mathematics Assessment

**DOI:** 10.3390/jintelligence14040070

**Published:** 2026-04-20

**Authors:** Huan Kuang

**Affiliations:** Department of Educational Psychology and Learning Systems, Florida State University, Tallahassee, FL 32306, USA; hkuang2@fsu.edu

**Keywords:** computational psychometrics, PISA, action sequences, expert-engineered features, process data, deep learning, neural networks, RNN, GRU, LSTM

## Abstract

Computer-based assessments generate rich process data that captures examinees’ interactions with test items. Using process data from the U.S. PISA 2012 computer-based mathematics assessment sample, this study applied recurrent neural networks to predict item-level correctness and assessment-level latent proficiency. The analysis also examines the impact of expert-engineered features, levels of architectural complexity, action variability, and score variability on model performance. At the item level, most models achieved AUC values around 0.80, indicating good predictive performance. Moderate correlations were observed between latent proficiency from 30 items and predictions based on process data from a subset of items (*n* = 10). For item-level models, adding expert-engineered features reduces training time and may improve predictive performance with low action variability. For the assessment-level models, adding expert-engineered features improved performance. Model complexity, including model type (i.e., standard RNN, GRU, and LSTM), number of nodes, and number of layers, had little effect on accuracy and efficiency. Moreover, items with greater action variability were associated with better model performance. The findings suggest that simple neural network architectures are sufficient for modeling process data with limited action variability and that combining action sequences with expert-engineered features improves accuracy, efficiency, and interpretability.

## 1. Introduction

The shift to computer-based assessments enables the systematic collection of detailed interaction records that document examinees’ behavioral traces during test taking. These records, often referred to as process data or log data, capture information such as clicks, keystrokes, and corresponding timestamps, providing rich insights into how students navigate tasks and engage with the assessments. Process data have attracted growing scholarly attention and have been analyzed for multiple purposes, including examining test-takers’ response strategies and behaviors (Wise, 2015; Ercikan et al., 2020; Rios et al., 2017; Wise & Kuhfeld, 2020), informing assessment design (Gao, 2021), and extending psychometric models (Wise & DeMars, 2006; Zhan et al., 2018).

One major line of research has used process data to explore test-takers’ behaviors during assessment. For example, process data has been shown to reveal patterns of disengagement and provide insights into their potential causes (Guo et al., 2016; Wise & Gao, 2017; Kuang & Sahin, 2023). Process data has also been used to identify item pre-knowledge (Pan & Wollack, 2023), cheating, and behaviors intended to game the system (Gurung et al., 2021). Such research helps strengthen test security and prevent test fraud (van der Linden & van Krimpen-Stoop, 2003; Chuang et al., 2017; Man et al., 2019). In addition, analyses of process data can illuminate how test-takers use tools like calculators and speech-to-text to solve problems (Liao & Sahin, 2020; Sahin, 2021), supporting evidence-based decisions about assessment accommodations.

Another major line of research examines the integration of process data into measurement models and ability estimation. It is motivated by the recognition that item responses alone do not capture all relevant information about examinees’ competencies. Several item response theory (IRT) models have incorporated response time as an additional source of information. For example, Wise and DeMars (2006) proposed an effort-moderated IRT model that accounts for rapid-guessing behavior to enhance the accuracy of item parameter estimates. Likewise, integrating process data has shown potential for improving adaptive assessments (Chang & Ying, 1996). Kern and Choe (2021) proposed a Joint Expected A Posteriori (J-EAP) estimator that incorporates response times as an additional source of information to estimate the latent trait in computerized adaptive testing, while controlling for differential speed. Process data has also been integrated into cognitive diagnosis models, with Zhan et al. (2018) introducing a joint noisy “and” gate (DINA) model that leverages both item responses and response actions to enhance the assessment of problem-solving competence. Zhan et al.’s (2018) findings suggested that including response actions improved the precision and accuracy of IRT parameters for a problem-solving item.

To integrate process data into statistical and machine-learning models, researchers have developed methods to transform unequal-length sequences into structured representations. A common approach involves constructing expert-engineered features, such as total response time (Schnipke & Scrams, 2002; Lee & Chen, 2011; Kyllonen & Zu, 2016; Chen & Cui, 2020), time spent on the first visit, number of actions (Sahin & Colvin, 2020; Kuang & Sahin, 2023), number of visits, and action frequencies. These features summarize key aspects of test-takers’ behavior and are commonly studied in educational data mining and computational psychometrics due to their interpretability. Prior research shows that expert-engineered features derived from process data provide insights into students’ problem-solving strategies, engagement, and performance. However, expert-engineered features may oversimplify complex process data by summarizing sequential behaviors into aggregated variables, leading to a loss of temporal and behavioral information and potentially limiting predictive performance.

In recent years, researchers have developed data-driven approaches for transforming process data. He and von Davier (2016) transformed action sequences into n-gram features by decomposing process data into contiguous subsequences of fixed length and encoding their occurrences as feature vectors. Building on this n-gram representation, Qiao and Jiao (2018) applied machine-learning models to analyze action sequences in problem-solving tasks, showing that n-gram features effectively distinguish successful from unsuccessful attempts. Tang et al. (2020) applied multidimensional scaling (MDS) to action sequences, producing a dissimilarity matrix based on pairwise comparisons of these sequences. Building on this work, Zhang et al. (2022) incorporated MDS-derived process features into latent ability estimation by jointly modeling these features with item responses, demonstrating improved measurement reliability. Their findings suggest that integrating process data is beneficial for low-stakes computer-based assessments, enabling shorter tests without compromising reliability. Xiong et al. (2024) proposed a sequential reservoir method (SRM) rooted in reservoir computing with an echo state network. Results from their simulation studies and empirical mathematics assessments demonstrate that the SRM effectively transforms action sequences into standardized and meaningful features, which enable the categorization of latent behavioral groups and the prediction of latent variables.

In addition to transformation approaches, many studies have used sequence and pattern mining approaches to directly model sequential features (Perera et al., 2008; Kinnebrew et al., 2013). Sequential features, which encode actions and corresponding timestamps as ordered sequences, could provide richer information about how response processes unfold. Process mining techniques such as alpha mining and fuzzy mining have been applied to analyze self-regulated learning processes (Bannert et al., 2014; Maldonado-Mahauad et al., 2018; Saint et al., 2022), collaborative learning patterns in project-based tasks (Reimann et al., 2009), as well as student engagement and course interaction dynamics in online learning platforms (Sedrakyan et al., 2016; Garcia et al., 2019; AlQaheri & Panda, 2022). Recently, process mining has also been used to examine problem-solving skills in assessment contexts by identifying and comparing common actions and action sequences (Ogut et al., 2024). However, pattern mining methods are fundamentally descriptive and primarily used to identify frequent behavioral patterns.

Beyond descriptive pattern mining, neural network architectures have been used for predictive modeling of sequential data. Cui et al. (2020) used an Adaptive Neuro-Fuzzy Inference System (ANFIS) model that combines fuzzy rules and a neural network to predict student problem-solving success using three expert-engineered features extracted from log data, and reported strong predictive performance. Unlike ANFIS, recurrent neural networks (RNNs) are designed to directly capture sequential dependencies in data without requiring extensive preprocessing or feature engineering, and have been widely applied to predict performance and learning outcomes from sequential data (Baniata et al., 2024; Li & Liu, 2021). Shin et al. (2022) further used RNNs to predict whether students needed additional tests based on previous scores and reported high prediction accuracy. These studies demonstrate the potential of RNNs for modeling action sequences in educational assessment.

Moreover, transformer-based models have been applied to process data. For example, transformer encoder models have been applied to log activity data from the Open University Learning Analytics Dataset to predict student performance at an early stage, achieving accuracy above 76% (Kusumawardani & Alfarozi, 2023). Similarly, a fine-tuned BERT model has been applied to action sequence data from three Problem-Solving in Technology-Rich Environments items in the 2012 PIAAC assessment to predict response accuracy, yielding promising results (Gorgun & Yildirim-Erbasli, 2026). However, compared to RNNs, transformer-based models require substantial computational resources and large training datasets. In addition, transformer architectures are generally more suitable for long sequences, and their advantages may be limited when applied to short sequences, where simpler models may be more efficient.

When modeling process data, researchers typically use either expert-engineered or sequential features. Although prior studies have demonstrated that both expert-engineered features and action sequences are important formats of process data, it remains unclear whether combining these two sources of information provides additional benefits, particularly in terms of predictive performance, modeling efficiency, and interpretability. To address this gap, this study investigates the joint modeling of action sequences and expert-engineered features using standard RNNs and their variants, including gated recurrent units (GRU) and long short-term memory (LSTM) networks, on data from the U.S. sample of the Program for International Student Assessment (PISA) 2012 computer-based mathematics assessment (CBMA). Specifically, this work explores the separate prediction of (a) item-level correctness and (b) assessment-level latent ability under two input conditions: using action sequences only and using action sequences combined with expert-engineered features.

This prediction framework is closely related to the broader literature on incorporating process data into measurement models and ability estimation. While previous studies have primarily focused on incorporating process data to improve parameter estimation and measurement precision within traditional measurement models, less attention has been given to directly predicting examinees’ competencies using process data. Because process data capture temporally ordered behaviors that occur before a response is submitted and may reflect examinees’ cognitive and strategic processes, they may provide valuable information for predicting and interpreting competency levels. Although some studies have explored prediction at the item level (Gorgun & Yildirim-Erbasli, 2026), little research has examined prediction at the assessment level, particularly whether actions from a subset of items can be used to predict proficiency estimates for the entire assessment. Such predictions could support adaptive testing, for example, by enabling early estimation of proficiency based on responses and actions from a small number of items. Therefore, this study aims to use process data to predict examinee competency at both the item and assessment levels.

Grounded in the computational psychometrics framework (von Davier, 2015, 2017; Mislevy, 2021), this study also aims to advance understanding of how different levels of architectural complexity (i.e., model types, number of nodes, and number of layers) influence predictive performance. Such an investigation is essential for evaluating the reliability of deep-learning approaches applied to educational measurement and for informing methodological strategies to address such reliability concerns. In addition, this study examined how model performance varies across items with different action variability (i.e., the average number of actions, the number of unique actions, and the proportion of predominant action), and score variability (i.e., proportion of predominant score category; see Section 2.7 for details). This analysis is important from the assessment perspective, as it provides insight into how differences in item design relate to model performance in educational measurement. These foundational perspectives motivated the following four main research questions (RQs) for this study:

RQ1: To what extent do expert-engineered features enhance the accuracy and efficiency of RNN models regarding predicting examinees’ competencies at item and assessment levels using data from the U.S. sample of the PISA 2012 CBMA?

RQ2: How do levels of architectural complexity influence the accuracy and efficiency of the RNN models regarding predicting examinees’ competencies at item and assessment levels using data from the U.S. sample of the PISA 2012 CBMA?

RQ3: What are the relationships between action variability and item-level predictive accuracy, as well as between score variability and item-level predictive accuracy, based on data from the U.S. sample of the PISA 2012 CBMA?

## 2. Materials and Methods

### 2.1. Data

This study used data from the PISA 2012 computer-based mathematics assessment (CBMA). PISA was developed by the Organisation for Economic Co-operation and Development (OECD) to assess student performance and provide comparative indicators of education systems worldwide (OECD, 2000). It is administered every three years to 15-year-old students in more than 70 countries in reading, mathematics, and science. In each cycle, one subject is assessed as the major domain, while the other two serve as minor domains for trend monitoring (OECD, 2013). In 2012, mathematics was the major domain, and PISA introduced CBMA, an optional computer-based mathematics assessment, for the first time. CBMA included interactive mathematics items that required students to engage with on-screen features such as graphs and tables. The dataset is publicly available and provides detailed item responses and action sequences (OECD, 2014), and access to the data is provided through the link in the Data Availability Statement of this study.

PISA 2012 CBMA dataset used in this study includes three units, namely CD Production (CM015), Star Points (CM020), and Body Mass Index (CM038). Each unit shares a common context and contains several items. For example, the CD Production unit (CM015) contains three items: CM015Q01, CM015Q02, and CM015Q03. Item names encode both the unit and the item number; for instance, CM015Q01 refers to item 1 in unit CM015 (Bardini, 2014). The original CM020Q01 item is provided in Figure S1 in the Supplementary Materials as an example. Unit CM020 in PISA 2012 CBMA contains four items (i.e., Q01, Q02, Q03 and Q04), and unit CM038 contains three items (i.e., Q03, Q05 and Q07). All items were administered within the same cluster, with each CBMA cluster allotted 20 min. The testing interface allowed test-takers to use a built-in calculator and access a formula page, as illustrated in Figure S2.

Data for this study were drawn from a sample of 402 U.S. students, including 188 females (47%) and 214 males (53%). Most students were in Grade 10 (*n* = 291), followed by Grades 11 (*n* = 68; see Table 1). In terms of birth year, 215 students (53%) were born in 1996, and 187 (47%) were born in 1997. Item-level sample sizes ranged from 378 to 402 students due to missing or invalid responses (see Table 2). Complete data across all ten items were available for 376 students (95%), which were used as the final sample for the assessment-level model.

### 2.2. Expert-Engineered Features

To address RQ1, six groups of expert-engineered features were extracted for each item: (1) total response time, (2) total time on each event, (3) time before the first attempt, (4) rapid response indicator, (5) total number of events, and (6) frequency of each event. A summary of these expert-engineered feature groups at the item level is provided in Table S1 in the Supplementary Materials. Approximately ten variables were extracted for each item, depending on the number of unique actions available for that item (see Table 2). For example, there are 12 item-level expert-engineered features for item CM015Q01. Two time-based variables—“total test response time” and “average response time”—across all ten items were extracted at the assessment level. In total, 103 expert-engineered features were extracted at the assessment level.

### 2.3. Sequential Features

Table 2 reports the sample size, number of expert-engineered features, and sequence length statistics (i.e., minimum, maximum, mean, and standard deviation) for each item and for all ten items combined, which are treated as assessment-level action sequences. In this study, sequential data were represented as sequences of events in temporal order. Because sequence lengths varied across examinees, the event sequences were padded with zeros to the maximum sequence length for each item to ensure equal-length input sequences. These padded sequences were then used as inputs to the RNN models. Therefore, the dimension of the sequential input is determined by the maximum sequence length for each item, as shown in Column 5 of Table 2. The dimension of the expert-engineered features corresponds to the number of expert-engineered features, which is reported in Column 3 of Table 2.

The steps below outline the preparation of sequential features:

- Denote the events variable as *A* = [*a*_1_, *a*_2_, …, *a_n_*], where each *a*_i_ represents an action taken at the *i*th time step.
- Assign a unique vector representation to each action. This set of predefined action vectors contains all unique actions, and is designated as *V* = {*v*_1_, *v*_2_, …, *v_m_*}, where *v_j_* represents the vector representation of the *j*th action. The size of each action vector is denoted as *d*, i.e., *v_j_* ∈ *ℝ^d^*.
- Initialize an empty resulting vector, denoted as *R* = [].
- Retrieve the vector representation of the current action a_i_ by mapping it to its corresponding action in vector *V*, with *v_i_* denoting the vector representation of the *i*th action in *A*.
- Append the vector representation *v*_i_ to the resulting vector *R* in temporal order. This specifically entails updating *R* as [*R*; *v*_i_].

To facilitate batch processing, which enables parallelization and enhances computational efficiency during training, padding was performed. First, the maximum length among all action sequences, denoted as *l*, was determined. If the length of the current resulting vector *V* was less than *l*, zero vectors were appended to the end of the sequence until its length equaled *l*.

### 2.4. Output Variables

For the item-level models, the outcome variables are the scored item responses (i.e., correctness). According to the PISA handbook, these scores are coded as either binary or categorical depending on the item type. Specifically, items CM015Q01 and CM038Q03 are binary items, whereas the remaining items are categorical. For the assessment-level models, the outcome variable is one of the plausible values (PVs). The PISA 2012 CBMA dataset provides five PVs that are strongly correlated with each other (lowest r = 0.92, *p* < .001). PVs are multiple imputations of student ability and are typically combined when making inferences about examinees’ proficiency levels (Jewsbury et al., 2024; Wu, 2005; OECD, 2014). In the current study, however, our primary objective was to compare the predictive performance of different neural network models rather than draw inferences about proficiency. Since all candidate models were trained and evaluated on the same fold splits and the same PV, restricting the analysis to one PV can be considered a fair comparison. Therefore, results based on a single plausible value are reported, following the approach used in previous studies such as Bulut et al. (2024). The selected plausible value (i.e., PV1) is continuous and approximately normally distributed, ranging from 200.99 to 753.26 (M = 498.37, SD = 88.98). It was normalized to have a mean of 0 and a standard deviation of 1 in this study. Moreover, it is important to note that these PVs are estimated with all 30 items from the full mathematics assessment rather than the 10 items included in the analytic dataset.

### 2.5. Description of Levels of Architectural Complexity

To address RQ2, levels of architectural complexity were defined from three dimensions: (a) the type of models, namely RNN, LSTM, and GRU; (b) the number of layers in the model; and (c) the number of nodes in the hidden layer.

#### 2.5.1. Type of Models

Standard RNN, GRU, and LSTM are well-suited for this purpose because they explicitly model temporal dependencies in sequential data, and their outputs have strong potential for integration with psychometric models. The standard RNN has the simplest structure, followed by GRU, while LSTM has the most complex architecture. RNN is designed for processing sequential data, with each recurrent unit that takes an input *x* and generates an output *h*. The hidden layer unit *h*^(*t*+1)^ at time *t* + 1 takes both the input *x*^(*t*+1)^ and the information from the previous hidden layer unit *h*^(*t*)^ into account. The mathematical representation of an RNN model is given by the following:

(1) $$\left\{\begin{array}{l} h^{\left(t\right)}=\;f\;\left(\alpha_{1}+Wh^{\left(t-1\right)}+Ux^{\left(t\right)}\right)\\ y^{\left(t\right)}=\alpha_{2}+Vh^{\left(t\right)} \end{array}\right.$$

where $x^{\left(t\right)}$, $h^{\left(t\right)}$, $y^{\left(t\right)}$ represents the input unit, hidden unit, and output unit at time *t*, respectively. The parameters $\alpha_{1}$ and $\alpha_{2}$ represent bias vectors (i.e., intercepts), *W* represents the weight matrices of the hidden-to-hidden connection, *U* represents the weight matrices of the input-to-hidden connection, *V* represents the weight matrices of the hidden-to-output connection, and *f*(·) represents a nonlinear activation function.

To examine more complex recurrent architectures, the LSTM model was included as an extension of the standard RNN. LSTMs include memory cells that give them a more intricate architecture. These cells serve as the repository for the network’s long-term memory, selectively storing or discarding information as needed. The mathematical representation of a vanilla LSTM model is as follows:

(2) $$\left\{\begin{array}{l} i^{\left(t\right)}=\sigma \left(W_{i}x^{\left(t\right)}+U_{i}h^{\left(t-1\right)}+\alpha_{i}\;\right)\\ f^{\left(t\right)}=\sigma \left(Wx^{\left(t\right)}+U_{f}h^{\left(t-1\right)}+\alpha_{f}\;\right)\\ o^{\left(t\right)}=\sigma \left(W_{o}x^{\left(t\right)}+U_{o}h^{\left(t-1\right)}+\alpha_{o}\right)\\ c^{\left(t\right)}=f^{\left(t\right)}\;⨀{\;c}^{\left(t-1\right)}+i_{t}\;⨀\;tanh(W_{c}x^{\left(t\right)}+U_{c}h^{\left(t-1\right)}+\alpha_{c})\\ h^{\left(t\right)}=o^{\left(t\right)}\;⨀\;tanh(c^{\left(t\right)})\\ y^{\left(t\right)}=\alpha_{y}+Vh^{\left(t\right)} \end{array}\right.$$

where the subscript *t* indicates the time, *W* represents the weight matrices of the input-to-hidden connection, *U* represents the weight matrices of the hidden-to-hidden connection, *V* represents the weight matrices of the hidden-to-output connection, and α represents the bias of the cell state. $x_{t}$ donates the input vector with the dimension of features equal to *m*, $i_{t}$ denotes the input gate vector ranging from 0 to 1, and $f_{t}$ denotes the forget gate vector ranging from 0 to 1. $o_{t}$ denotes the out gate vector ranging from 0 to 1, $h_{t}$ denotes the hidden state vector ranging from −1 to 1, $c_{t}$ denotes the cell state vector ranging from −1 to 1, and ⨀ represents element-wise multiplication.

GRU was included as another architecture that provides a simpler alternative to LSTM while still addressing the limitations of standard RNNs. With fewer parameters, GRU is easier to train and computationally more efficient. Unlike LSTM, GRU does not use a separate cell state; instead, the hidden state acts as the network memory and is controlled by two gates: the reset gate and the update gate. The mathematical representation of a GRU model is given by the following:

(3) $$\left\{\begin{array}{l} z^{\left(t\right)}=\sigma \left(W_{z}x^{\left(t\right)}+U_{z}h^{\left(t-1\right)}+\alpha_{z}\;\right)\\ r^{\left(t\right)}=\sigma \left(W_{r}x^{\left(t\right)}+U_{r}h^{\left(t-1\right)}+\alpha_{r}\right)\\ \tilde{h^{\left(t\right)}}=tanh(W_{h}x^{\left(t\right)}+U_{h}(r^{\left(t\right)}⊙h^{\left(t-1\right)})+\alpha_{h})\\ h^{\left(t\right)}=(1-z^{\left(t\right)})\;⨀\;h^{\left(t-1\right)}\;+-z^{\left(t\right)}\;⨀\;\tilde{h^{\left(t\right)}}\\ y^{\left(t\right)}=\alpha_{y}+Vh^{\left(t\right)} \end{array}\right.$$

where the subscript *t* indicates the time, *W* represents the weight matrices of the input-to-hidden connection, *U* represents the weight matrices of the hidden-to-hidden connection, *V* represents the weight matrices of the hidden-to-output connection, and α represents the bias of the cell state. $x_{t}$ donates the input vector with the dimension of features equal to *m*, $z_{t}$ denotes the update gate vector ranging from 0 to 1, and $r_{t}$ denotes the reset gate vector ranging from 0 to 1. $\tilde{h^{\left(t\right)}}$ denotes the candidate hidden state vector ranging from −1 to 1, $h_{t}$ denotes the hidden state vector, and ⨀ represents element-wise multiplication.

#### 2.5.2. Number of Layers

In addition to model type, levels of architectural complexity were also manipulated by varying the number of hidden layers in the networks. In RNN models, input data is transformed as it passes through the hidden layers, and each additional hidden layer enables the model to learn more complex and higher-level representations of the input data. Increasing the number of layers is assumed to enhance the model’s representational capacity. However, it also raises the risk of overfitting and increases the computational cost during training. In this study, two configurations for the layer are compared: single-layer architecture and double-layer architecture. These configurations were chosen based on the size of the PISA 2012 CBAM dataset.

#### 2.5.3. Number of Nodes

Levels of architectural complexity were further manipulated by varying the number of nodes in the hidden layer. Each hidden layer consists of nodes that process input data and pass useful information. The number of nodes influences a neural network’s ability to capture complex relationships; more nodes generally increase model complexity by increasing the number of learnable parameters. However, a large number of nodes does not guarantee better performance and may lead to overfitting, reducing generalization to unseen data. In this study, two configurations for the node were used: 50 and 100 nodes for predicting latent abilities at the assessment level, and 10 and 20 nodes for predicting correctness at the item level.

### 2.6. Model Evaluation

When evaluating the model performance in terms of predictive accuracy, it is common practice to use multiple metrics to obtain a comprehensive understanding (Bosch & Paquette, 2018). In this study, assessment-level continuous predictions were evaluated using Pearson correlation and Root Mean Squared Error (RMSE). Meanwhile, item-level categorical correctness was evaluated using Area Under the Curve (AUC) and Cohen’s Kappa. Moreover, efficiency is an important consideration alongside model performance, particularly in relation to levels of architectural complexity (Bishop, 2006). In this study, average training time across the five cross-validation folds was reported as a measure of model efficiency.

In addition to performance and efficiency, model interpretability was also examined using SHapley Additive exPlanations (SHAP). SHAP is based on Shapley values from cooperative game theory. It explains model predictions by assigning each feature a contribution value that represents its impact on the prediction relative to a baseline value. In this study, SHAP was used to quantify feature importance and to interpret how sequential and expert-engineered features contributed to the model predictions.

### 2.7. Action Variability and Score Variability

In educational measurement contexts, action and score variability may influence predictive performance, as lower variability in response patterns and score distributions may increase the difficulty of prediction tasks for the models. By comparing model performance across items with different levels of action and score variability, this study aims to better understand the factors that influence predictive performance, which may have implications for assessment design. To address RQ3, action variability is operationalized using three dimensions, and score variability is operationalized using one dimension.

- The average number of actions per item. It was calculated by summing all recorded actions for the item and dividing by the total number of test-takers ($\frac{N_{all\;actions}}{N_{all\;test-takers}}$), with higher values indicating greater action variability. For example, an item that requires examinees to complete an average of three actions may reflect a more straightforward response process. In contrast, an item that requires an average of ten actions may reflect a more involved response process with greater variability in action sequences.
- The number of unique actions. For a given item, it was computed by counting all distinct actions associated with the item ($\sum_{i=1}^{n}i$, where *i* index each of the unique actions), with higher values indicating greater item complexity. For example, an item that contains three unique actions may provide relatively limited action options, whereas an item with ten unique actions may allow for greater variability in action sequences.
- The proportion of the predominant action. For an item, it was computed in three steps. First, the frequency of each unique action was calculated. Second, the most frequently occurring action was identified. Third, its count was divided by the total number of recorded actions ($\mathrm{proportion}=\frac{N_{\mathrm{most}\;\mathrm{frequent}\;\mathrm{action}}}{N_{\mathrm{all}\;\mathrm{actions}}}$). This measure serves as an indicator of action variability, with higher values indicating lower item complexity, as most test-takers exhibit the same action. For example, a value of 0.5 indicates that 50% of the recorded actions correspond to the most frequent action.
- The proportion of the predominant score category. For an item, it was also computed in three steps. First, the frequency of each unique scoring category was calculated. Second, the most frequently occurring scoring category was identified. Third, its count was divided by the total number of test-takers ($\mathrm{proportion}=\frac{N_{\mathrm{most}\;\mathrm{frequent}\;\mathrm{score}}}{N_{\mathrm{test}\;\mathrm{takers}}}$). This measure serves as an indicator of score variability, with higher values indicating lower item complexity, as most test-takers received the same score. For example, a value of 1 indicates that all examinees received the same score for the item.

### 2.8. Implementation Details

This section presents the cross-validation procedure and the fundamental components of RNNs, LSTMs, and GRUs, including activation functions, loss functions, optimizers, and hyperparameters.

#### 2.8.1. Cross-Validation

A five-fold stratified cross-validation approach was used to address label imbalance. At the item level, stratification ensured that each fold contained approximately the same proportions of each correctness category as in the full dataset. At the assessment level, stratification was performed based on quantiles of the latent ability. Performance measures obtained from the five folds were averaged to produce an overall performance estimate.

To prevent student-level data leakage, cross-validation splitting was performed at the student level rather than the sequence level, meaning that all action sequences from a given student were assigned to the same fold. Student ID was used as the grouping variable to ensure that no student appeared in both the training and validation sets.

#### 2.8.2. Activation Function

In RNNs, the hyperbolic tangent (tanh) function is used as the activation function for the hidden state and is defined as follows:

(4) $$tanh=\;f\left(x\right)=\;\frac{2}{1+\exp\left(-2x\right)\;}$$

In GRUs and LSTMs, the tanh function is used for both the cell state and the hidden state, while the sigmoid function is applied to the input, forget, and output gates. The sigmoid function is defined as follows:

(5) $$t=\;f\left(x\right)=\;\frac{1}{1+\exp\left(-x\right)}$$

#### 2.8.3. Loss Function

Binary cross-entropy loss, also known as log loss, was used for binary classification tasks, specifically to predict binary correctness for items CM015Q01 and CM038Q03. Categorical cross-entropy loss was applied to items with more than two categories of correctness. At the assessment level, latent ability as a continuous variable was modeled using the Mean Squared Error loss function.

#### 2.8.4. Model Training and Optimization

The Adam optimizer was used for all models because it adaptively adjusts learning rates and demonstrates fast convergence during training. The initial learning rate for all models was set to 0.0001 and was dynamically updated by the Adam algorithm during training. As a result, the effective learning rates may differ across models and training iterations due to Adam’s adaptive learning rate mechanism.

**Figure 1:**
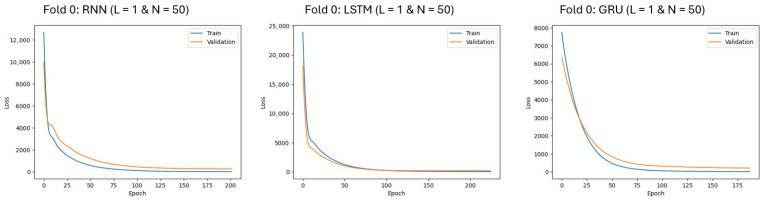
Assessment-level Loss Curves for One-layer Models with 50 Nodes (1st fold).

Models were trained for up to 10,000 epochs using early stopping and a patience parameter of 5. The 10,000 represents the maximum number of training epochs. In practice, training typically stopped much earlier (approximately 50–200 epochs in this study) once the validation loss stopped improving. The patience parameter was selected based on empirical performance, meaning that training stopped if the validation loss did not improve for five consecutive epochs. Training and validation loss curves were monitored to evaluate model learning and ensure that early stopping prevented overfitting. Figure 1 shows the loss curves for the assessment-level model in the first fold for one-layer RNN, LSTM, and GRU models with 50 nodes. All RNN, LSTM, and GRU models were implemented using the *TensorFlow* library (version 2.6.0) with Python 3.10.9.

## 3. Results

### 3.1. Contribution of Expert-Engineered Features at Assessment Level

To address RQ1, which examines the contribution of integrating expert-engineered features, we conducted a comparative analysis between models using only sequential features and models using both sequential and expert-engineered features. The expert-engineered features were added as covariates at the output (dense) layer, allowing them to contribute to the final prediction while preserving the sequential representations learned by the network models.

#### 3.1.1. Assessment Level Correlations

Overall, the predicted latent ability exhibited a moderate positive correlation with the PISA plausible value, with correlation coefficients ranging from 0.28 to 0.62 (see Table 3). It is worth noting that these correlations were between PV from 30 items with predictions based on actions from a subset of ten items, suggesting that competencies may be approximated at an early stage with a relatively small number of items.

Adding expert-engineered features improved model performance across all 12 model architectures (3 types of model × 2 numbers of layers × 2 numbers of nodes). The largest improvement was observed for the two-layer LSTM model with 100 nodes, where the inclusion of expert-engineered features increased the correlation from 0.36 to 0.62. The one-layer RNN model with 50 nodes showed the smallest improvement, with the correlation increasing from 0.30 to 0.34.

#### 3.1.2. Assessment Level RMSEs

In terms of RMSEs, the inclusion of expert-engineered features is generally associated with lower prediction errors across most model configurations (see Table 4). The largest decrease was observed for the one-layer RNN model with 100 nodes, where RMSE decreased from 1.16 to 0.93. However, two exceptions were observed for the GRU models with 100 nodes (both one-layer and two-layer), where RMSE showed a slight increase (from 0.99 to 1.03 and from 1.04 to 1.05, respectively).

#### 3.1.3. Assessment Level Training Time

In terms of efficiency, the inclusion of expert-engineered features at the assessment level increased training time across all conditions (see Table 5). For example, in the simplest RNN configuration with a single layer and 50 nodes, training time increased from 46.7 s with only sequential features to 130.1 s with expert-engineered features. Similarly, for an LSTM model with a single layer and 50 nodes, training time increased from 94.3 to 1189.32 s.

#### 3.1.4. Assessment Level SHAP Values

**Figure 2:**
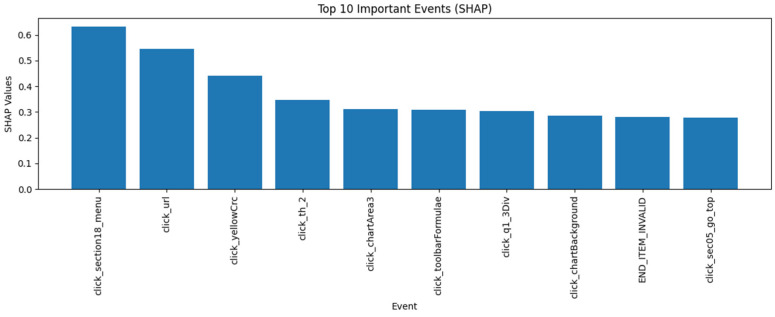
Top Ten Events Ranked by Mean SHAP Values with Sequential Input Only.

Figure 2 shows the ten most important events at the assessment level, identified based on mean SHAP values using only sequential features. They were primarily navigation and interaction behaviors, such as clicking menu sections, URLs, and toolbar functions. Events related to chart interaction and invalid item endings were also among the most influential features. These results suggest that students’ interaction behaviors, navigation patterns, and tool usage played an important role in predicting latent ability.

**Figure 3:**
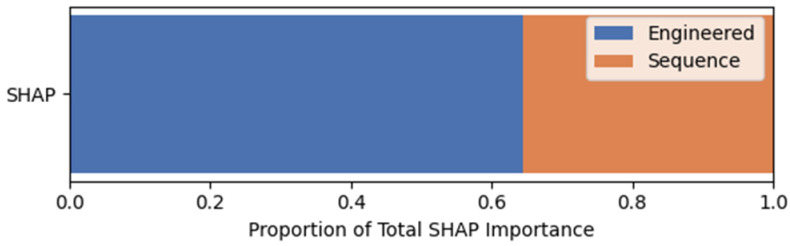
Relative Contribution of Sequential and Expert-Engineered Features.

Figure 3 shows the relative contributions of the two feature types based on SHAP values when both sequential and expert-engineered features were used at the assessment level. The contribution was calculated as the proportion of the total absolute SHAP values attributed to each feature type. Expert-engineered features accounted for approximately 64% of the total SHAP importance. Meanwhile, sequential features accounted for approximately 36%, indicating that the model relied more heavily on expert-engineered features for prediction at the assessment level.

**Figure 4:**
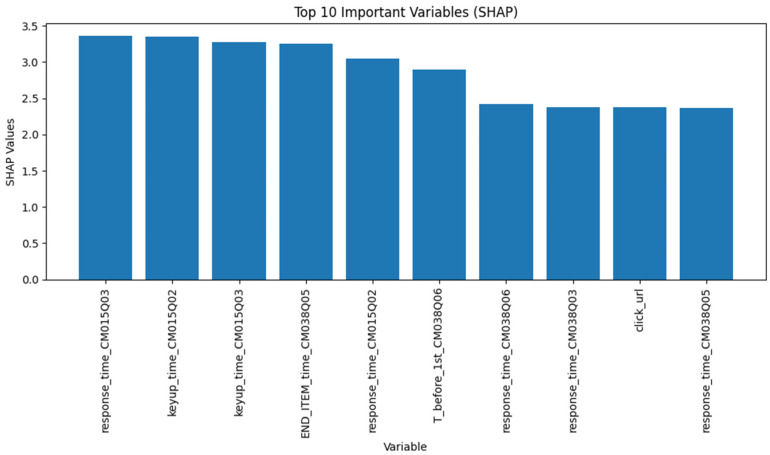
Top Ten Variables Ranked by Mean SHAP Values with Both Features.

Figure 4 presents the ten most important variables, based on mean SHAP values, for both sequential and expert-engineered features at the assessment level. The majority of the top ten important variables were time-based features, including response times for items CM015Q02, CM015Q03, CM038Q03, CM038Q05, and CM038Q06, as well as time for “keyup” action for items CM015Q02 and CM015Q03. Only one sequential feature, “click_url”, was identified as one of the top ten important variables.

### 3.2. Contribution of Expert-Engineered Features at Item Level

#### 3.2.1. Item Level AUC

**Figure 5:**
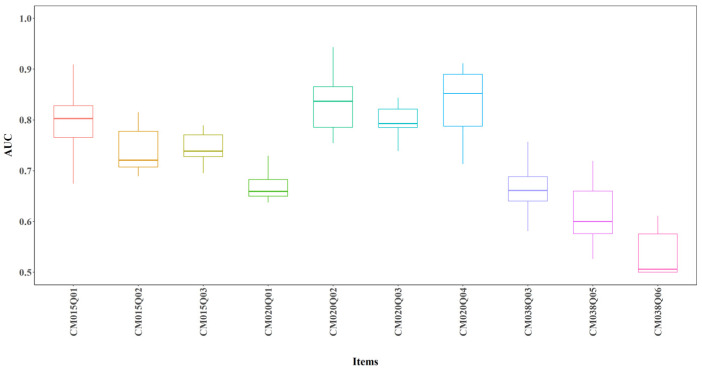
AUC by Item for All Models.

Overall, predicted correctness showed good agreement with observed true correctness, regardless of whether expert-engineered features were included. Figure 5 shows boxplots of AUC across 24 model configurations (3 model types × 2 layer configurations × 2 node configurations × 2 input feature sets) for each item. Colors represent different models, with one model per item. The average AUC exceeded 0.6 for most items, indicating good predictive performance and the ability to distinguish between correct and incorrect responses. One exception was CM038Q06, which showed average AUC around 0.5 (see Figure 5).

**Figure 6:**
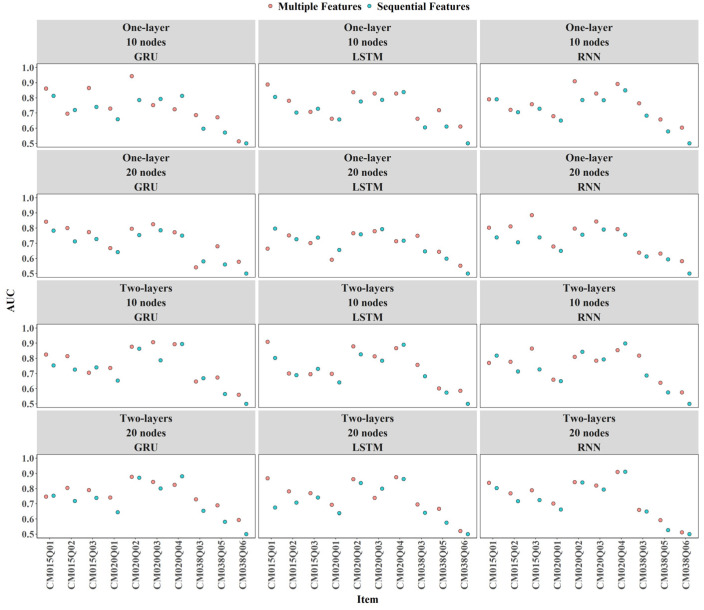
Contribution of Expert-Engineered Features to AUC.

Incorporating expert-engineered features into item-level predictions resulted in slightly higher AUC values across most conditions, as shown by the red dots (see Figure 6). However, there were some exceptions where AUC decreased slightly, for example, a two-layer LSTM model with 20 nodes for items CM020Q02.

#### 3.2.2. Item Level Kappa

**Figure 7:**
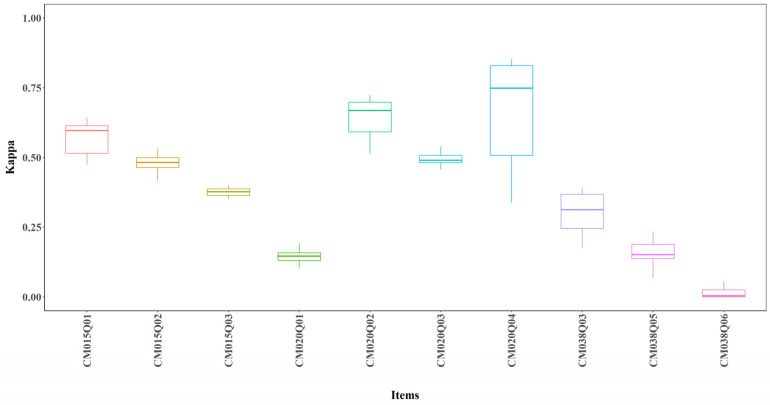
Kappa by Item for All Models.

Figure 7 shows boxplots of Kappa across 24 model configurations for each item. The average Cohen’s Kappa exceeded 0.25 for most items, indicating performance substantially better than random guessing. One exception was CM038Q06, which showed an average Kappa of around 0 (see Figure 7).

**Figure 8:**
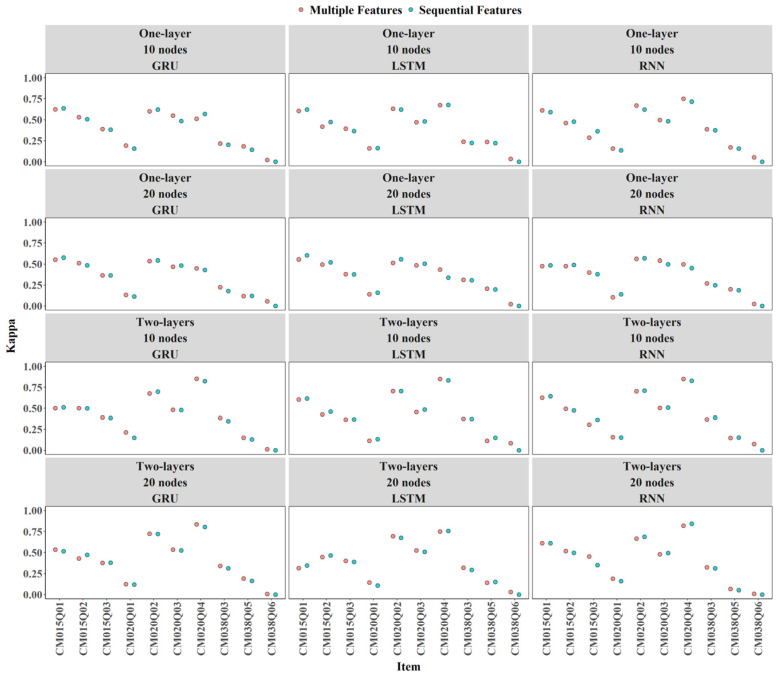
Contribution of Expert-Engineered Features to Kappa.

Incorporating expert-engineered features improved Kappa values across most conditions, though the increases were minor. In some cases, Kappa decreased slightly. For example, the two-layer LSTM with 10 nodes for CM038Q05 (see Figure 8).

#### 3.2.3. Item Level Training Time

**Figure 9:**
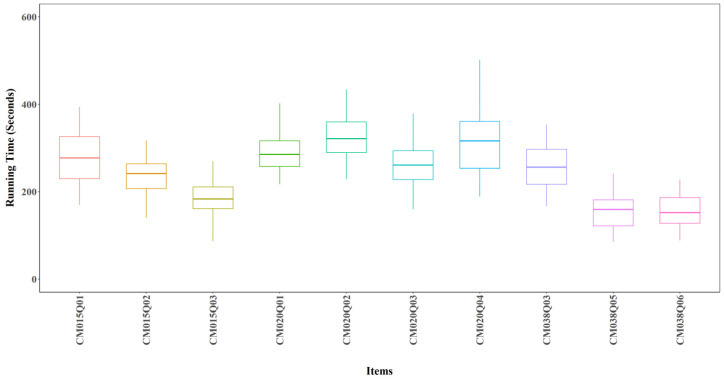
Training time by Item for All Models.

Figure 9 shows boxplots of training time across 24 model configurations for each item. On average, each model takes about 5 min (300 s), though there is some variability. CM015Q03, CM038Q05, and CM038Q06 consistently showed short training times across all configurations.

**Figure 10:**
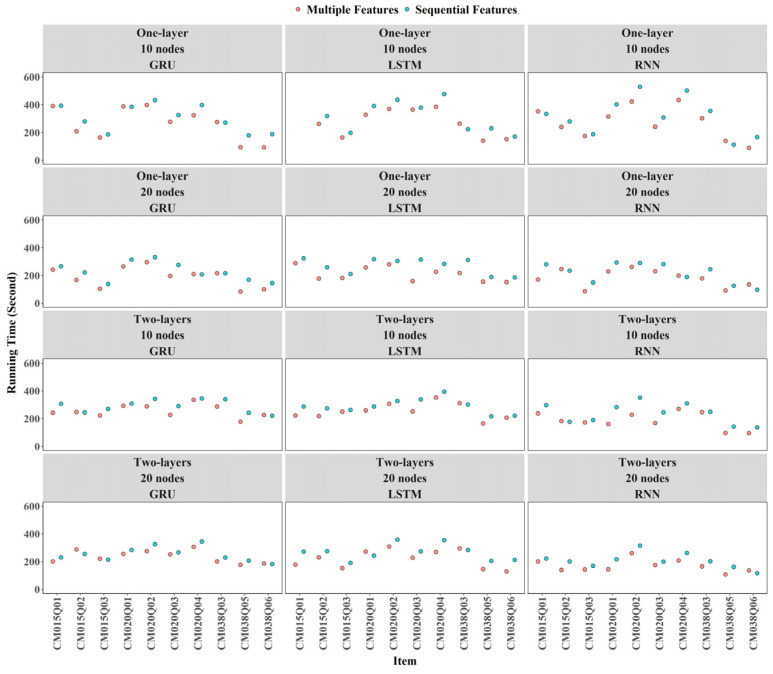
Contribution of Expert-Engineered Features to Training Time.

The results indicate that incorporating expert-engineered features improves computational efficiency (Figure 10). Across most items and configurations, models trained with both expert-engineered and sequential features took less time while achieving comparable performance, as discussed in the AUC and Cohen’s Kappa parts. For models relying only on sequential features, average training times were 308 s across ten items, whereas models that included expert-engineered features generally converged in 267 s on average.

#### 3.2.4. Item Level SHAP Values

**Figure 11:**
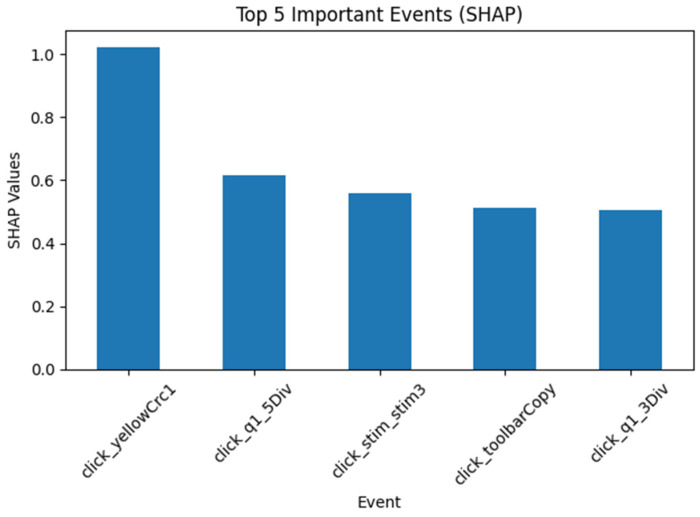
Top Five Events for CM015Q01 with Sequential Input Only.

Item CM015Q01 was used as an example to report item-level SHAP values. The patterns were similar across most items. Figure 11 shows the five most important variables based on mean SHAP values when only sequential features were used for CM015Q01. All of them were specific click events.

**Figure 12:**
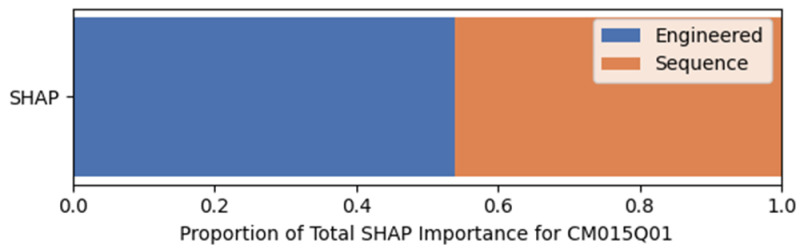
Relative Contribution of Sequential and Expert-Engineered Features for CM015Q01.

Figure 12 shows the relative contribution of sequential and expert-engineered features based on SHAP values for CM015Q01, when both features were used. Sequential features accounted for approximately 53% of total SHAP importance, compared to 47% for expert-engineered features, indicating greater reliance on sequential features for prediction.

**Figure 13:**
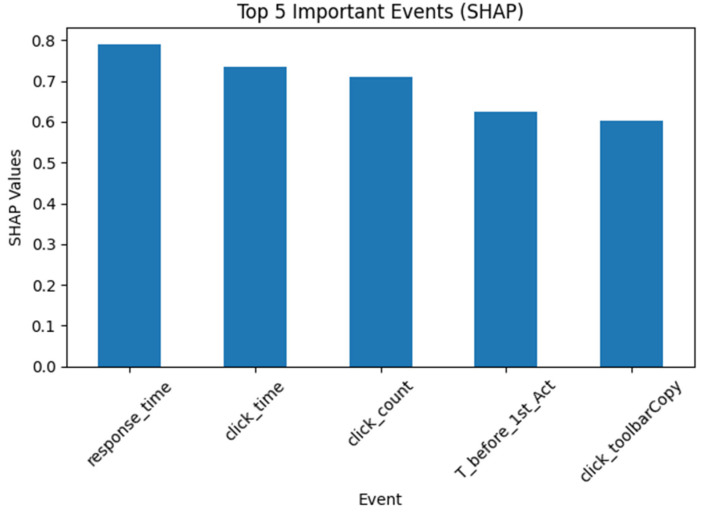
Top Five Variables for CM015Q01 with Both Features.

Figure 13 presents the five most important variables, based on mean SHAP values, for CM015Q01 using both sequential and expert-engineered features. Four of the top five features were expert-engineered (see Figure 12). Three of these were time-based features, namely total response time, time on click actions, and time before the first action. One additional expert-engineered feature was the total number of actions. The remaining feature was sequential; specifically, clicking the copy button on the toolbar.

### 3.3. Results for Different Levels of Architectural Complexity

To address RQ2, which examines how levels of architectural complexity influence model performance and efficiency, we focused on models using only sequential features. This allowed us to rule out the impact of the expert-engineered features discussed previously.

#### 3.3.1. Assessment Level

As presented in Table 3, the correlation between predicted latent ability at the assessment level and the PISA plausible values was moderately positive (Benesty et al., 2009). When only sequential features were used, the correlation ranged from 0.28 to 0.41. Overall, LSTM models consistently performed better than or as well as both RNN and GRU models, except for the two-layer model with 100 nodes. In terms of RMSE (Table 4), no discernible differences were observed across the 12 models (3 model types × 2 numbers of layers × 2 numbers of nodes) using sequential features. RMSE values for the RNN models ranged from 1.09 to 1.17, from 0.99 to 1.06 for GRU models, and from 1.06 to 1.1 for LSTM models.

There was no clear relationship between model performance and the number of layers or the number of nodes. Model performance was relatively similar across different configurations. Among the four LSTM models using sequential features (2 layer configurations × 2 node configurations), the two-layer LSTM with 50 nodes achieved the highest correlation (*r* = 0.41). This was followed by the single-layer LSTM with 100 nodes (*r* = 0.37), the two-layer LSTM with 100 nodes (*r* = 0.35), and the one-layer LSTM with 50 nodes (*r* = 0.34). Taken together with the RMSE results, these findings indicate that increasing the number of layers and nodes does not yield meaningful performance improvements for the PISA 2012 CBMA at the assessment level for the U.S. sample.

Across all tested conditions, RNN models consistently required shorter training times than GRU and LSTM models. Increasing the number of layers and nodes in the RNN models led to a marginal increase in training time of approximately ten seconds. Training times for RNN models ranged from 46.7 to 88.9 s, whereas training times for GRU models ranged from 97.03 to 728.72, and for LSTM models ranged from 94.34 to 1976.11 s (see Table 5).

#### 3.3.2. Item Level

**Figure 14:**
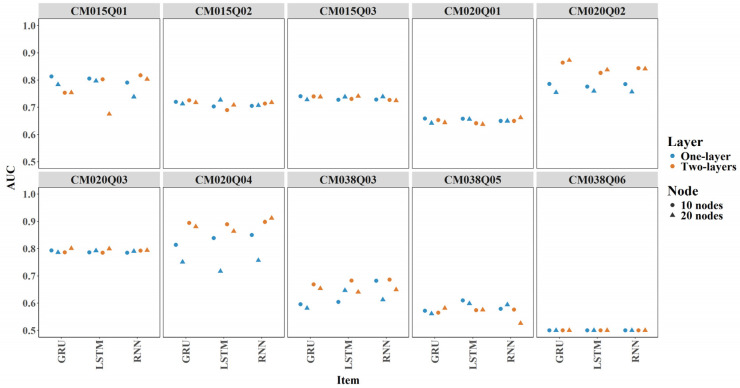
Comparison of Models by Item-Level AUC Scores.

**Figure 15:**
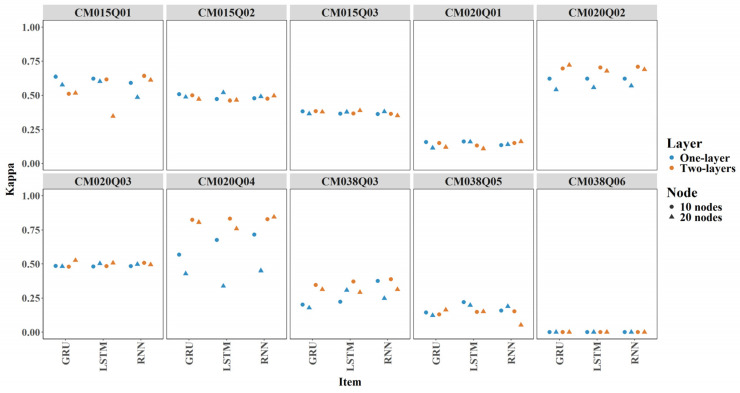
Comparison of Models by Item-Level Kappa Scores.

Overall, there were only minor differences among the RNN, GRU, and LSTM models in terms of AUC and Cohen’s Kappa (see Figure 14 and Figure 15). For items CM020Q02, CM020Q04, and CM038Q03, increasing numbers of layers and nodes tended to improve AUC and Kappa. For other items, adding additional layers or nodes did not result in meaningful performance gains.

**Figure 16:**
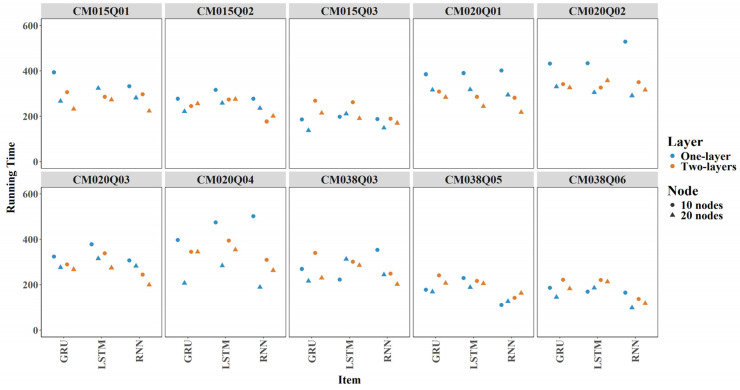
Comparison of Models by Training Time.

At the item level, training time was very similar across RNN, GRU, and LSTM models. For most items, increasing the number of nodes or layers in RNN, LSTM, or GRU models was not associated with substantial differences in training time (see Figure 16).

### 3.4. Model Performance in Relation to Action and Score Variability

Table 6 summarizes the action and score variability measures for each item and shows substantial differences across items. For example, CM015Q03 had the highest average number of actions (28), indicating longer action sequences, whereas CM038Q05 and CM038Q06 had a much lower average number of actions (2), indicating shorter response processes.

In terms of the number of unique actions, most items have about 10 unique actions. CM038Q03 had a very large number of unique actions (80), while CM038Q05 and CM038Q06 had only two unique actions. Specifically, CM038Q05 and CM038Q06 were fill-in-the-blank items in which all test-takers followed identical action sequences, with no actions recorded between START_ITEM and END_ITEM.

The proportion of the predominant action varied across items, indicating different levels of action variability. Items CM015Q03, CM020Q01, and CM020Q03 showed relatively high proportions of the predominant action (68–72%), suggesting that most test-takers performed similar actions on these items. In contrast, items such as CM020Q02 (23.19%) and CM038Q03 (9.69%) showed much lower proportions, indicating greater variability in action sequences and more diverse response behaviors. Items CM038Q05 and CM038Q06 showed a predominant action proportion of 50% because they had only two actions, both used equally often.

The proportion of the predominant score category also varied across items, ranging from 43% (CM015Q03) to 73% (CM015Q02), indicating that none of the items showed extremely skewed score distributions (i.e., values close to 0 or 1). In summary, items differ considerably in their action sequences and score distributions.

To address RQ3, the corresponding associations are presented graphically, with model performance (i.e., AUC and Cohen’s Kappa) shown against (a) the average number of actions, (b) the total number of unique actions, (c) the variance of actions, and (d) the variance of item responses. For clarity, only results from the two-layer, 20-node LSTM model with multiple inputs are shown in the plots.

#### 3.4.1. Average Number of Actions and Model Performance

**Figure 17:**
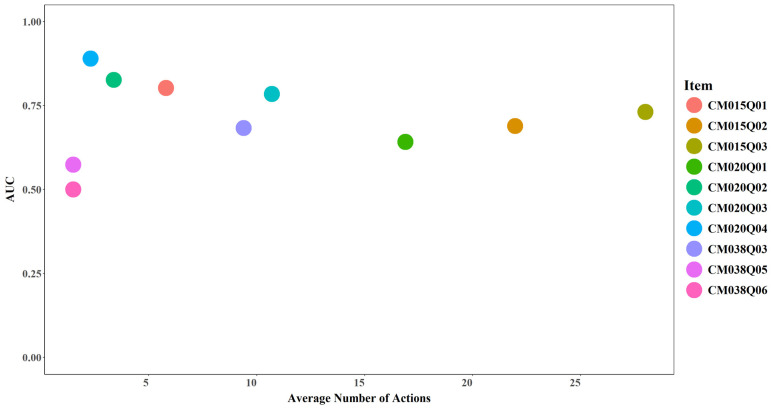
AUC by Average Number of Actions for Each Item.

**Figure 18:**
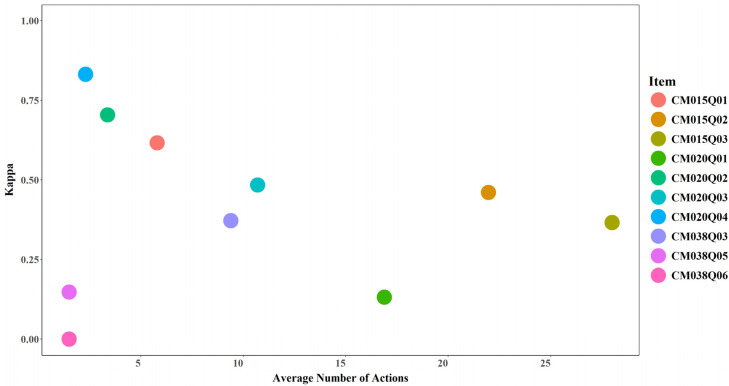
Kappa by Average Number of Actions for Each Item.

No clear relationship was observed between the average number of actions and model performance. Increases in the average number of actions did not necessarily correspond to higher AUC or Cohen’s Kappa values (see Figure 17 and Figure 18). However, an average of two actions appears to represent a critical threshold, beyond which model performance exceeds random prediction (AUC > 0.5 and Cohen’s Kappa > 0). For instance, CM038Q05, which recorded 2 actions on average, achieved an AUC slightly above 0.5 and a kappa around 0.15 after incorporating expert-engineered features. CM038Q06, which also recorded 2 actions on average, achieved an AUC of 0.5 and a kappa of 0 even after incorporating expert-engineered features.

#### 3.4.2. Unique Actions and Model Performance

**Figure 19:**
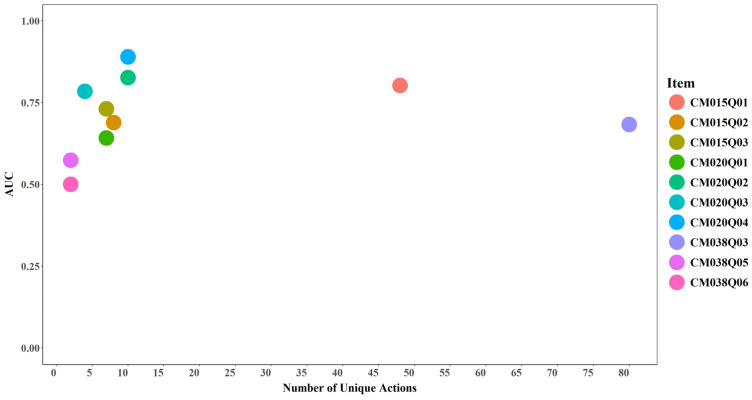
AUC by Number of Unique Actions for Each Item.

**Figure 20:**
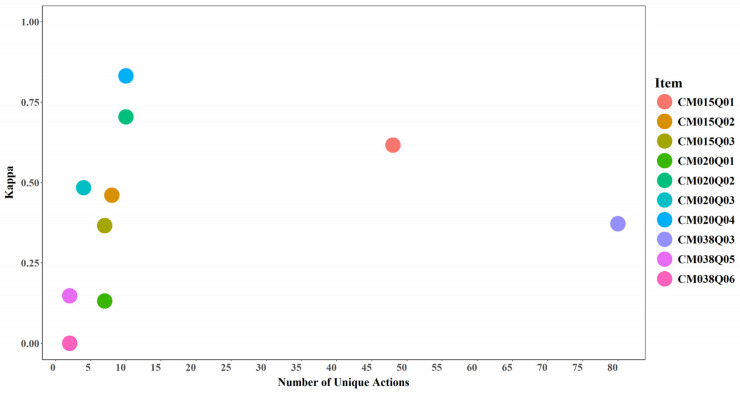
Kappa by Number of Unique Actions for Each Item.

Despite the small number of items (*n* = 10), the results suggest a trend whereby items with a greater number of unique actions enable the model to better distinguish among score categories. Models required more than two unique actions to achieve meaningful predictive performance (AUC > 0.5 and Cohen’s Kappa > 0). Items with fewer than two unique actions—such as CM038Q05 and CM038Q06—did not provide sufficiently informative action sequences for learning meaningful patterns (see Figure 19 and Figure 20).

#### 3.4.3. Proportion of the Predominant Action and Model Performance

The proportion of the predominant action reflects the extent to which test-takers took a single dominant action when interacting with an item. Higher values indicate that most test-takers used the same action, suggesting lower action variability. Lower values indicate greater diversity in actions and higher action variability. No clear pattern was observed between the proportion of the predominant action and model performance in this study. In other words, action dominance by itself is insufficient to explain model performance.

**Figure 21:**
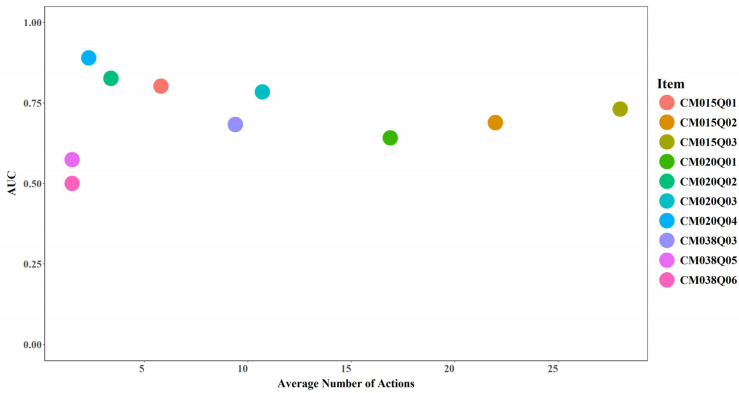
AUC by Proportion of the Predominant Action for Each Item.

**Figure 22:**
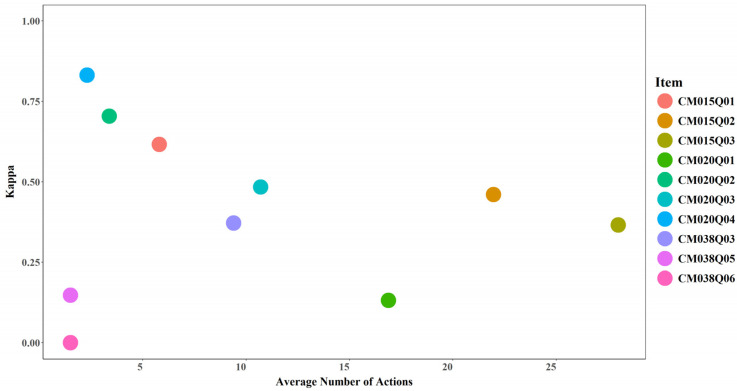
Kappa by Proportion of the Predominant Action for Each Item.

For items CM015Q01, CM015Q02, and CM015Q03, the most frequent action was “keyup” with an event value of “q1NoCopy”. The corresponding proportions were 0.37, 0.37, and 0.70, respectively (Figure 21 and Figure 22). “ACER_EVENT” was the predominant action for CM020Q01 (0.68) and CM020Q03 (0.72). “Click” was the predominant action for CM020Q04 (0.30). “END_ITEM” was the predominant action for CM020Q02 (0.23) and CM038Q03 (0.30). For CM038Q05 and CM038Q06, actions were evenly split between “START_ITEM” and “END_ITEM” (0.50 each), and the action sequences for these two items exhibited no variance.

#### 3.4.4. Proportion of Predominant Score Category and Model Performance

**Figure 23:**
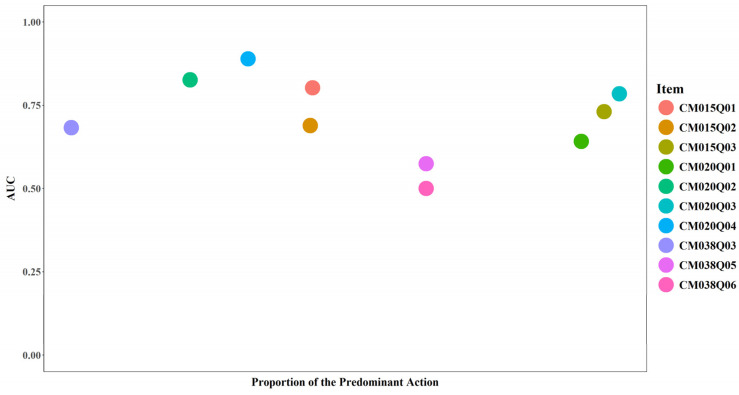
AUC by Proportion of the Predominant Score Category for Each Item.

**Figure 24:**
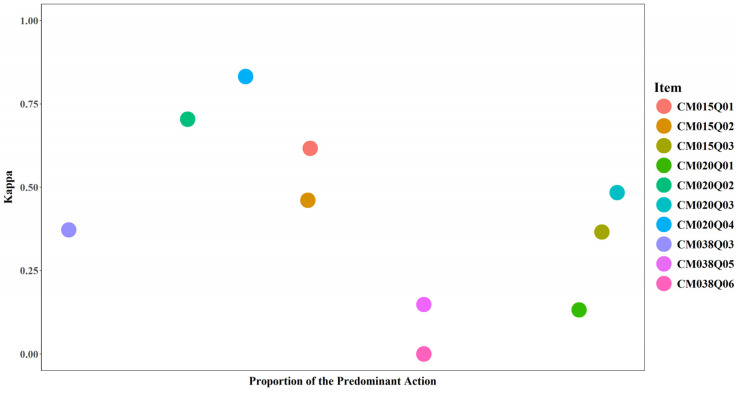
Kappa by Proportion of the Predominant Score Category for Each Item.

The proportion of the predominant score category reflects the degree to which scores are concentrated in a single category for an item. For example, a value of 0.50 indicates that 50% of students received the most frequent score. Higher values indicate that most test-takers received the same score, suggesting lower score variability and lower item discrimination, whereas lower values indicate greater score variability across categories. Overall, no clear pattern was observed between the proportion of the predominant score category and model performance in this study (Figure 23 and Figure 24), suggesting that the proportion of the predominant score category alone does not meaningfully explain model performance for the PISA 2012 CBMA with the U.S. sample.

For four items (CM015Q01, CM015Q02, CM020Q02, and CM038Q03), the majority of test-takers received a “full score”, with proportions of 0.55, 0.73, 0.48, and 0.64, respectively. In contrast, for six items (CM015Q03, CM020Q01, CM020Q03, CM020Q04, CM038Q05, and CM038Q06), the predominant score category was “no score”, with proportions of 0.43, 0.53, 0.71, 0.55, 0.67, and 0.74, respectively.

## 4. Discussion

With respect to RQ1, we examined the extent to which expert-engineered features enhance the accuracy and efficiency of RNNs, GRUs, and LSTMs for predicting examinees’ competencies using data from the U.S. PISA 2012 CBMA sample. The results suggest that integrating expert-engineered features improved model performance at the assessment level, although it increased training time. For item-level models, integrating expert-engineered features did not improve model performance but reduced training time. Moreover, in scenarios where there is little or no variance in test-takers’ actions, including expert-engineered features, have the potential to improve model performance (i.e., CM038Q05). These findings are consistent with prior research in educational data mining and computational psychometrics, which demonstrated that process data can provide meaningful predictive information and that expert-engineered features can improve model interpretability (Baker et al., 2016; von Davier, 2017).

With respect to RQ2, we examined different RNN architectures by varying the RNN type (i.e., standard RNN, GRU, and LSTM), the number of layers, and the number of nodes per layer. The results indicate that, at both the assessment and item levels, simpler neural network architectures (i.e., standard RNNs) with fewer layers and nodes achieved predictive performance and efficiency similar to those of more complex architectures. On one hand, the length and diversity of item-level action sequences are somewhat limited. For eight of the ten items, there are fewer than ten unique actions. For items CM015Q01 and CM038Q03, although there are 48 and 80 unique actions, respectively, the average sequence lengths are only approximately 5 and 10, and many possible sequences are rarely observed. At the assessment level, we only have action sequences from 10 items to predict latent proficiency from 30 items. This indicates that the action sequences in this data may not be sufficiently long or diverse to fully support more complex models. On the other hand, this may also be due to the relatively small sample size in the PISA 2012 CBMA data from the United States sample (Anderson, 1960). When datasets with larger sample sizes, a larger number of unique actions, and longer item-level sequence lengths become available, it would be worthwhile to conduct a systematic investigation (e.g., a learning-curve analysis) of sample size and average as well as unique actions for more complex models.

With respect to RQ3, we examined the association between action variability and item-level predictive accuracy, as well as between score variability and item-level predictive accuracy. The results indicate that items requiring only minimal interaction tend to provide limited behavioral variation for modeling. Test questions that require students to do more than just click once (e.g., drag or move through multiple steps) may be more useful, as these interactive actions can provide more detailed information about how students think and solve the problem (Gamire & Pearson, 2006; DiCerbo, 2014). When the data allow, it may also be beneficial to further differentiate actions by aspect, such as item-, tool-, and system-related actions. Such categorization may help reflect different solution approaches. For example, in mathematics items, item-related actions may include interactions essential to answering test items, such as selecting an option in a multiple-choice question or submitting a response. Tool-related actions may include interactions that are not strictly required but can support task completion, such as the use of a calculator or a highlighter. System-related actions may refer to interactions with the testing platform that are not directly tied to answering test items, such as changing the page theme or color scheme. The 2012 PISA CBMA dataset includes only item-related actions. The absence of tool-related and system-related information limits the incorporation of potentially informative expert-engineered features, such as the number of visits, tool usage, and answer changes. Future research could further examine how different types of actions are associated with students’ performance, since such work may help educators better understand students’ mathematical problem-solving processes.

Moreover, given the unique characteristics of the PISA 2012 CBMA data from the U.S. sample, these findings may not directly generalize to PISA math assessments in other countries or regions. The model based on the U.S. data may reflect specific cultural and curricular characteristics that differ from those in other countries or regions. In addition, for some items, the PISA interfaces vary across countries and contexts, and some tools (e.g., calculators) may function slightly differently (Bardini, 2014). Likewise, these findings should not be directly generalized to other mathematics assessments or other subject areas, because the required actions and the underlying problem-solving processes may differ.

This study makes several theoretical, methodological, and practical contributions to research on process data. From a theoretical perspective, the study shows that action variability is related to model predictive accuracy, highlighting the importance of considering the distribution of action sequences when modeling process data. From a methodological perspective, this study provides a systematic framework for combining action sequences and expert-engineered features when predicting competence. Integrating expert-engineered features with sequence models may improve model interpretability and, in some cases, reduce training time while maintaining predictive performance. These two types of information should therefore not be treated as an either–or choice, but rather modeled jointly as complementary sources of information. From a practical perspective, the findings have implications for item design and process data collection. Items that involve more interactive actions and multi-step processes may provide richer process data that better capture students’ problem-solving behaviors. The findings also indicate that action sequence data from a small number of items can be used to predict latent ability estimates obtained from a larger item pool. This has important implications for adaptive testing, as process data may support more accurate item selection during the initial stages of the test, when response-based ability estimates are still unstable.

## 5. Limitations and Future Research

This study has several limitations and can be extended in several ways. Firstly, given the unique characteristics of the PISA 2012 CBMA and the U.S. sample, the findings may not generalize to other assessments or populations. Future research should examine other datasets, such as the National Assessment of Educational Progress (NAEP), the Program for the International Assessment of Adult Competencies (PIAAC), and the Trends in International Mathematics and Science Study (TIMSS), to validate and further examine the findings of this study.

Secondly, this study does not examine the minimum sample size required to effectively implement RNN models at either the item or assessment level. Future work should more directly examine the trade-offs among sample size, action distribution, and model complexity using learning-curve analysis. Data augmentation or simulation studies could also be conducted to determine minimum sample size requirements and provide practical recommendations.

Thirdly, this study used identical model structures at the item level. While this uniform approach ensures consistency, it may not produce the optimal architecture for every item, as effective model structures can vary across items. Exploring additional model configurations, including different numbers of layers and nodes for each item, may improve predictive performance and provide better insight into model behavior.

Lastly, levels of architectural complexity were reflected by the model type and hyperparameters (i.e., the number of layers and nodes). Because the focus of this study was not architectural optimization, we did not include a separate analysis of the number of trainable parameters. Future research could further examine the parameter-to-sample-size ratio to guide model complexity relative to available data.

## 6. Conclusions

Overall, this study contributes to the field of computational psychometrics by demonstrating the potential of deep-learning models to simultaneously model both sequential and expert-engineered features to predict examinees’ competency at the item and assessment levels using data from the U.S. PISA 2012 CBMA sample. The findings suggest that including expert-engineered features can be beneficial, particularly for reducing training time and improving the interpretability of sequential features. By integrating domain knowledge into machine-learning models, expert-engineered features can make model outputs more transparent and easier to refine, thereby supporting more meaningful analytical interpretations (Amershi et al., 2014; Mosqueira-Rey et al., 2023). Regarding architectural complexity, for similar sample sizes and action variability, relatively simple neural network architectures may serve as a starting point for modeling process data. In practice, researchers may benefit from starting with a simpler architecture before introducing additional complexity.

We encourage future studies to investigate action and score variability using the four measures proposed in this study and to consider additional measures, including interactions among them. The findings also highlight the value of interactive assessment items (Gamire & Pearson, 2006), as they generate richer process data and provide deeper insights into examinees’ cognitive processes and competencies. To fully leverage these data, systematic strategies and frameworks are needed to capture and store log data when developing computer-based assessments or intelligent systems. Action and score variability may be an important direction for future research, as it may help identify the minimum level of interaction required for an item to generate sufficient behavioral variation for prediction. Overall, the methodological framework proposed in this study is transferable and may be applied in comparable research contexts, thereby providing a foundation for future investigations.

## Figures and Tables

**Table 1:** Student Grade Levels in the Sample.

Grade	Frequency	Percentage
8	1	0.24%
9	41	10.20%
10	291	72.39%
11	68	16.92%
12	1	0.24%

**Table 2:** Summary of Expert-engineered Features and Sequence Lengths.

Unit	Sample Size	Number of Expert Features	Minimum Sequence Length	Maximum Sequence Length	Average Sequence Length	SD for Average Sequence Length
CM015Q01	402	12	2	42	5.80	5.26
CM015Q02	401	10	2	153	21.97	21.44
CM015Q03	401	10	2	163	28.00	26.56
CM020Q01	400	12	2	135	16.88	15.78
CM020Q02	399	10	2	22	3.38	2.51
CM020Q03	397	12	2	96	10.70	10.14
CM020Q04	395	10	2	12	2.30	0.95
CM038Q03	393	10	2	55	9.39	8.81
CM038Q05	383	8	2	2	2	0
CM038Q06	378	7	2	2	2	0
Ten Items	376	103	8	313	99.41	52.86

**Table 3:** Correlation Coefficients for All Models at Assessment Level.

	Sequential Features			Multiple Features ^c^		
	RNN	GRU	LSTM	RNN	GRU	LSTM
L ^a^ 1 N ^b^ 50	0.30	0.36	0.34	0.34	0.53	0.56
L1 N100	0.30	0.33	0.37	0.51	0.47	0.55
L2 N50	0.38	0.33	0.41	0.48	0.44	0.54
L2 N100	0.28	0.34	0.36	0.45	0.42	0.62

^a^ L refers to the number of layers, ^b^ N refers to the number of nodes, and ^c^ Multiple Features refers to the combination of sequences and expert-engineered features.

**Table 4:** RMSE for All Models at Assessment Level.

	Sequential Features			Multiple Features ^c^		
	RNN	GRU	LSTM	RNN	GRU	LSTM
L ^a^ 1 N ^b^ 50	1.09	1.01	1.07	1.00	0.90	0.99
L1 N100	1.16	0.99	1.06	0.93	1.03	1.00
L2 N50	1.11	1.06	1.09	1.01	0.89	0.89
L2 N100	1.17	1.04	1.10	0.97	1.05	0.89

^a^ L refers to the number of layers, ^b^ N refers to the number of nodes, and ^c^ Multiple Features refers to the combination of sequences and expert-engineered features.

**Table 5:** Training Time (Seconds) for All Models at Assessment Level.

	Sequential Features			Multiple Features ^c^		
	RNN	GRU	LSTM	RNN	GRU	LSTM
L ^a^ 1 N ^b^ 50	46.77	97.03	94.34	130.09	179.25	1189.32
L1 N100	73.51	134.71	99.92	140.78	201.15	1198.59
L2 N50	72.83	124.64	1976.11	184.68	683.80	2002.03
L2 N100	88.94	728.72	934.59	142.89	816.33	2054.72

^a^ L refers to the number of layers, ^b^ N refers to the number of nodes, and ^c^ Multiple Features refers to the combination of sequences and expert-engineered features.

**Table 6:** Summary of Action and Score Variability Measures by Item.

Item	Average Number of Actions	Number of Unique Actions	Proportion of Predominant Action	Proportion of Predominant Score Category
CM015Q01	5.80	48	37.09%	55%
CM015Q02	21.97	8	36.83%	73%
CM015Q03	28.00	7	70.18%	43%
CM020Q01	16.88	7	67.61%	53%
CM020Q02	3.38	10	23.19%	48%
CM020Q03	10.70	4	71.92%	71%
CM020Q04	2.30	10	29.77%	55%
CM038Q03	9.39	80	9.69%	64%
CM038Q05	2	2	50%	67%
CM038Q05	2	2	50%	74%

## Data Availability

The data analyzed in the current study are publicly available from the PISA public database, which can be found at https://www.oecd.org/en/data/datasets/pisa-2012-cba-database.html (accessed on 26 March 2026).

## Supplementary Materials

The following supporting information can be downloaded at https://www.mdpi.com/article/10.3390/jintelligence14040070/s1. Figure S1. Screenshot of CM020Q01; Figure S2. The Available Tools in the 2012 PISA CBAM: (A) Calculator and (B) Formulae page; Table S1. Item-level Expert-engineered Features; Table S2. AUC, Kappa, and Training Time for RNN Models with Sequential Features; Table S3. AUC, Kappa, and Training Time for LSTM Models with Sequential Features; Table S4. AUC, Kappa, and Training Time for GRU Models with Sequential Features; Table S5. AUC, Kappa, and Training Time for RNN Models with Multiple Features; Table S6. AUC, Kappa, and Training Time for LSTM Models with Multiple Features; Table S7. AUC, Kappa, and Training Time for GRU Models with Multiple Features.

## Abbreviations

The following abbreviations are used in this manuscript:

2PL	Two-Parameter Logistic
AUC	Area Under the Curve
CBAM	Computer-Based Assessment of Mathematics
DINA	Deterministic Input, Noisy “and” Gate
GPCM	Generalized Partial Credit Model
GRU	Gated Recurrent Units
IRT	Item Response Theory
J-EAP	Joint Expected A Posteriori Estimator
LSTM	Long Short-term Memory
NAEP	National Assessment of Educational Progress
PIAAC	Programme for the International Assessment of Adult Competencies
PISA	Programme for International Student Assessment
ReLU	Rectified Linear Unit
RNN	Recurrent Neural Network
RMSE	Root Mean Squared Error
SD	Standard Deviation
SRM	Sequential Reservoir Method
Tanh	Hyperbolic Tangent
