# Supplementary material for: Evaluating Neural Networks Architectures for Competency Prediction from Process Data Using PISA Computer-Based Mathematics Assessment
Source: J Intell. 2026 Apr 20;14(4):70. doi: 10.3390/jintelligence14040070 (PMC13118180; doi:10.3390/jintelligence14040070)
Supplement: Supplementary file 1 [file jintelligence-14-00070-s001.zip › jintelligence-4136128-supplementary.pdf]

Supplementary Materials

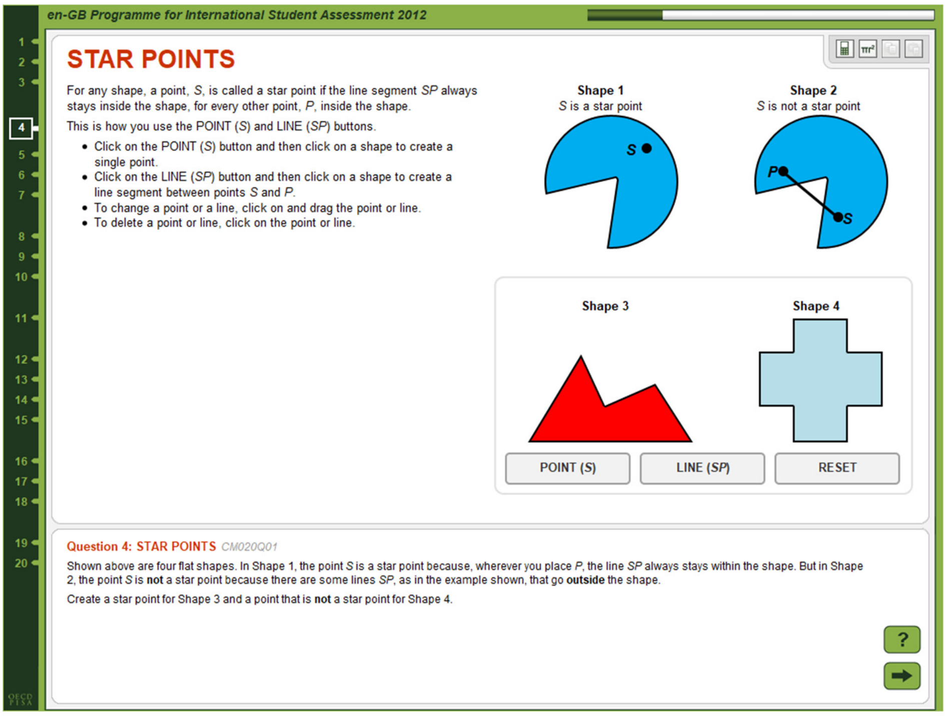

Figure S1. Screenshot of CM020Q01.

A

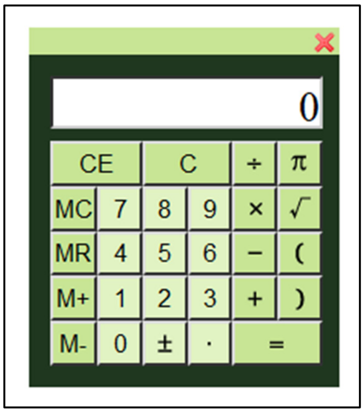

B

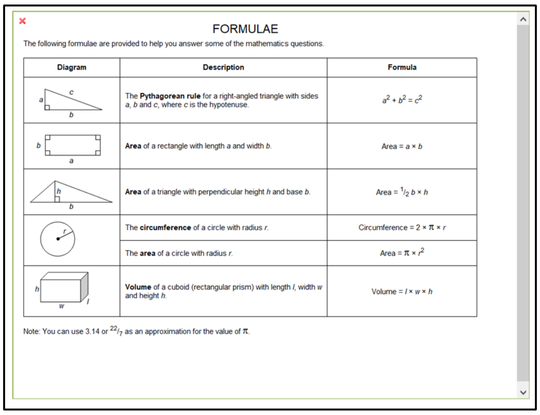

Figure S2. The Available Tools in the 2012 PISA CBAM: (A) Calculator and (B) Formulae page.

Table S1. Item-level Expert-engineered Features

No.	Variables	Types	Description
1	Total Response Time	Numerical	Time-based: time spent by an examinee on each item in the test, accounting for any potential multiple visits
2	Rapid Response Indicator	Binary	Time-based: 1, if the total response time for an examinee was shorter than 10% of the average total response time; 0, otherwise
3	Time before the First Attempt[1]	Numerical	Time-based: time spent by an examinee before his/her first attempt
4	Time on Each Event	Numerical	Time-based: time spent by an examinee on each event
5	Total Number of Events	Numerical	Action-based: the number of events each student had before exiting an item, accounting for any potential multiple visits
6	Count of Each Event	Numerical	Action-based: the number of events each student had in each event

Table S2. AUC, Kappa, and Training Time for RNN Models with Sequential Features

Item	Model	Layer	Node	Input	AUC	Kappa	Time
CM015Q01	RNN	1	10	sequence	0.79	0.59	332.84
CM015Q01	RNN	1	20	sequence	0.74	0.48	281.19
CM015Q01	RNN	2	10	sequence	0.82	0.64	296.70
CM015Q01	RNN	2	20	sequence	0.80	0.61	222.94
CM015Q02	RNN	1	10	sequence	0.70	0.48	277.63
CM015Q02	RNN	1	20	sequence	0.71	0.49	235.05
CM015Q02	RNN	2	10	sequence	0.71	0.48	177.09
CM015Q02	RNN	2	20	sequence	0.72	0.50	200.47
CM015Q03	RNN	1	10	sequence	0.73	0.36	187.18
CM015Q03	RNN	1	20	sequence	0.74	0.38	149.06
CM015Q03	RNN	2	10	sequence	0.73	0.36	189.48
CM015Q03	RNN	2	20	sequence	0.72	0.35	170.08
CM020Q01	RNN	1	10	sequence	0.65	0.13	401.84
CM020Q01	RNN	1	20	sequence	0.65	0.14	294.19
CM020Q01	RNN	2	10	sequence	0.65	0.15	281.47
CM020Q01	RNN	2	20	sequence	0.66	0.16	217.52
CM020Q02	RNN	1	10	sequence	0.79	0.62	528.76
CM020Q02	RNN	1	20	sequence	0.76	0.57	290.39
CM020Q02	RNN	2	10	sequence	0.84	0.71	350.47
CM020Q02	RNN	2	20	sequence	0.84	0.69	315.73
CM020Q03	RNN	1	10	sequence	0.78	0.48	306.72
CM020Q03	RNN	1	20	sequence	0.79	0.50	282.07
CM020Q03	RNN	2	10	sequence	0.79	0.51	245.05
CM020Q03	RNN	2	20	sequence	0.79	0.49	199.57
CM020Q04	RNN	1	10	sequence	0.85	0.71	501.73
CM020Q04	RNN	1	20	sequence	0.76	0.45	188.83
CM020Q04	RNN	2	10	sequence	0.90	0.83	309.39
CM020Q04	RNN	2	20	sequence	0.91	0.84	262.85
CM038Q03	RNN	1	10	sequence	0.68	0.38	353.86
CM038Q03	RNN	1	20	sequence	0.61	0.25	244.39
CM038Q03	RNN	2	10	sequence	0.69	0.39	249.06
CM038Q03	RNN	2	20	sequence	0.65	0.31	201.95
CM038Q05	RNN	1	10	sequence	0.50	0.00	110.46
CM038Q05	RNN	1	20	sequence	0.50	0.00	125.92
CM038Q05	RNN	2	10	sequence	0.50	0.00	142.40
CM038Q05	RNN	2	20	sequence	0.50	0.00	162.63
CM038Q06	RNN	1	10	sequence	0.50	0.00	165.41
CM038Q06	RNN	1	20	sequence	0.50	0.00	98.23
CM038Q06	RNN	2	10	sequence	0.50	0.00	136.63
CM038Q06	RNN	2	20	sequence	0.50	0.00	117.82

Table S3. AUC, Kappa, and Training Time for LSTM Models with Sequential Features

Item	Model	Layer	Node	Input	AUC	Kappa	Time
CM015Q01	LSTM	1	10	sequence	0.81	0.62	5323.74
CM015Q01	LSTM	1	20	sequence	0.80	0.60	323.73
CM015Q01	LSTM	2	10	sequence	0.80	0.62	285.93
CM015Q01	LSTM	2	20	sequence	0.67	0.35	272.69
CM015Q02	LSTM	1	10	sequence	0.70	0.47	316.89
CM015Q02	LSTM	1	20	sequence	0.73	0.52	258.38
CM015Q02	LSTM	2	10	sequence	0.69	0.46	273.78
CM015Q02	LSTM	2	20	sequence	0.71	0.46	275.46
CM015Q03	LSTM	1	10	sequence	0.73	0.36	197.59
CM015Q03	LSTM	1	20	sequence	0.74	0.38	210.34
CM015Q03	LSTM	2	10	sequence	0.73	0.37	261.80
CM015Q03	LSTM	2	20	sequence	0.74	0.39	190.59
CM020Q01	LSTM	1	10	sequence	0.66	0.16	390.71
CM020Q01	LSTM	1	20	sequence	0.66	0.16	317.98
CM020Q01	LSTM	2	10	sequence	0.64	0.13	285.85
CM020Q01	LSTM	2	20	sequence	0.64	0.11	243.65
CM020Q02	LSTM	1	10	sequence	0.78	0.62	434.18
CM020Q02	LSTM	1	20	sequence	0.76	0.56	305.18
CM020Q02	LSTM	2	10	sequence	0.83	0.70	326.78
CM020Q02	LSTM	2	20	sequence	0.84	0.68	357.34
CM020Q03	LSTM	1	10	sequence	0.79	0.48	378.64
CM020Q03	LSTM	1	20	sequence	0.79	0.50	314.73
CM020Q03	LSTM	2	10	sequence	0.78	0.48	338.18
CM020Q03	LSTM	2	20	sequence	0.80	0.51	273.88
CM020Q04	LSTM	1	10	sequence	0.84	0.68	475.38
CM020Q04	LSTM	1	20	sequence	0.72	0.34	283.27
CM020Q04	LSTM	2	10	sequence	0.89	0.83	394.53
CM020Q04	LSTM	2	20	sequence	0.86	0.76	353.84
CM038Q03	LSTM	1	10	sequence	0.60	0.22	222.81
CM038Q03	LSTM	1	20	sequence	0.65	0.31	312.39
CM038Q03	LSTM	2	10	sequence	0.68	0.37	300.67
CM038Q03	LSTM	2	20	sequence	0.64	0.29	284.84
CM038Q05	LSTM	1	10	sequence	0.50	0.00	229.34
CM038Q05	LSTM	1	20	sequence	0.50	0.00	188.01
CM038Q05	LSTM	2	10	sequence	0.50	0.00	217.07
CM038Q05	LSTM	2	20	sequence	0.50	0.00	205.39
CM038Q06	LSTM	1	10	sequence	0.50	0.00	169.56
CM038Q06	LSTM	1	20	sequence	0.50	0.00	185.88
CM038Q06	LSTM	2	10	sequence	0.50	0.00	221.34
CM038Q06	LSTM	2	20	sequence	0.50	0.00	213.07

Table S4. AUC, Kappa, and Training Time for GRU Models with Sequential Features

Item	Model	Layer	Node	Input	AUC	Kappa	Time
CM015Q01	GRU	1	10	sequence	0.81	0.64	393.51
CM015Q01	GRU	1	20	sequence	0.78	0.58	266.62
CM015Q01	GRU	2	10	sequence	0.75	0.51	306.56
CM015Q01	GRU	2	20	sequence	0.75	0.52	232.02
CM015Q02	GRU	1	10	sequence	0.72	0.51	277.38
CM015Q02	GRU	1	20	sequence	0.71	0.49	220.98
CM015Q02	GRU	2	10	sequence	0.73	0.50	244.86
CM015Q02	GRU	2	20	sequence	0.72	0.47	255.62
CM015Q03	GRU	1	10	sequence	0.74	0.38	185.74
CM015Q03	GRU	1	20	sequence	0.73	0.36	137.79
CM015Q03	GRU	2	10	sequence	0.74	0.38	269.10
CM015Q03	GRU	2	20	sequence	0.74	0.38	213.64
CM020Q01	GRU	1	10	sequence	0.66	0.16	384.98
CM020Q01	GRU	1	20	sequence	0.64	0.11	315.87
CM020Q01	GRU	2	10	sequence	0.65	0.15	308.51
CM020Q01	GRU	2	20	sequence	0.64	0.12	284.08
CM020Q02	GRU	1	10	sequence	0.79	0.62	432.38
CM020Q02	GRU	1	20	sequence	0.75	0.54	330.40
CM020Q02	GRU	2	10	sequence	0.86	0.70	342.28
CM020Q02	GRU	2	20	sequence	0.87	0.72	326.42
CM020Q03	GRU	1	10	sequence	0.79	0.49	324.04
CM020Q03	GRU	1	20	sequence	0.79	0.48	275.62
CM020Q03	GRU	2	10	sequence	0.79	0.48	289.08
CM020Q03	GRU	2	20	sequence	0.80	0.53	267.14
CM020Q04	GRU	1	10	sequence	0.81	0.57	396.71
CM020Q04	GRU	1	20	sequence	0.75	0.43	207.23
CM020Q04	GRU	2	10	sequence	0.89	0.82	345.02
CM020Q04	GRU	2	20	sequence	0.88	0.80	344.67
CM038Q03	GRU	1	10	sequence	0.60	0.20	269.45
CM038Q03	GRU	1	20	sequence	0.58	0.18	216.13
CM038Q03	GRU	2	10	sequence	0.67	0.35	340.04
CM038Q03	GRU	2	20	sequence	0.65	0.31	229.52
CM038Q05	GRU	1	10	sequence	0.50	0.00	177.97
CM038Q05	GRU	1	20	sequence	0.50	0.00	168.60
CM038Q05	GRU	2	10	sequence	0.50	0.00	241.52
CM038Q05	GRU	2	20	sequence	0.50	0.00	206.95
CM038Q06	GRU	1	10	sequence	0.50	0.00	186.63
CM038Q06	GRU	1	20	sequence	0.50	0.00	144.86
CM038Q06	GRU	2	10	sequence	0.50	0.00	221.61
CM038Q06	GRU	2	20	sequence	0.50	0.00	182.42

Table S5. AUC, Kappa, and Training Time for RNN Models with Multiple Features

Item	Model	Layer	Node	Input	AUC	Kappa	Time
CM015Q01	RNN	1	10	multiple	0.79	0.61	351.75
CM015Q01	RNN	1	20	multiple	0.80	0.47	169.90
CM015Q01	RNN	2	10	multiple	0.77	0.63	237.94
CM015Q01	RNN	2	20	multiple	0.84	0.61	201.41
CM015Q02	RNN	1	10	multiple	0.72	0.46	238.66
CM015Q02	RNN	1	20	multiple	0.81	0.48	245.95
CM015Q02	RNN	2	10	multiple	0.78	0.49	182.12
CM015Q02	RNN	2	20	multiple	0.77	0.52	140.16
CM015Q03	RNN	1	10	multiple	0.76	0.29	173.83
CM015Q03	RNN	1	20	multiple	0.88	0.40	86.24
CM015Q03	RNN	2	10	multiple	0.86	0.30	172.22
CM015Q03	RNN	2	20	multiple	0.79	0.45	144.24
CM020Q01	RNN	1	10	multiple	0.68	0.16	314.14
CM020Q01	RNN	1	20	multiple	0.68	0.10	228.55
CM020Q01	RNN	2	10	multiple	0.66	0.16	161.11
CM020Q01	RNN	2	20	multiple	0.70	0.19	144.49
CM020Q02	RNN	1	10	multiple	0.91	0.67	422.51
CM020Q02	RNN	1	20	multiple	0.80	0.56	262.30
CM020Q02	RNN	2	10	multiple	0.81	0.70	228.79
CM020Q02	RNN	2	20	multiple	0.84	0.67	261.34
CM020Q03	RNN	1	10	multiple	0.83	0.50	240.80
CM020Q03	RNN	1	20	multiple	0.84	0.54	229.94
CM020Q03	RNN	2	10	multiple	0.78	0.50	168.27
CM020Q03	RNN	2	20	multiple	0.82	0.48	174.93
CM020Q04	RNN	1	10	multiple	0.89	0.75	433.52
CM020Q04	RNN	1	20	multiple	0.79	0.50	198.01
CM020Q04	RNN	2	10	multiple	0.85	0.85	268.49
CM020Q04	RNN	2	20	multiple	0.91	0.82	207.66
CM038Q03	RNN	1	10	multiple	0.76	0.39	300.49
CM038Q03	RNN	1	20	multiple	0.64	0.27	178.39
CM038Q03	RNN	2	10	multiple	0.82	0.37	245.31
CM038Q03	RNN	2	20	multiple	0.66	0.32	166.75
CM038Q05	RNN	1	10	multiple	0.66	0.17	138.90
CM038Q05	RNN	1	20	multiple	0.63	0.20	92.65
CM038Q05	RNN	2	10	multiple	0.64	0.15	96.87
CM038Q05	RNN	2	20	multiple	0.59	0.07	109.17
CM038Q06	RNN	1	10	multiple	0.60	0.05	88.39
CM038Q06	RNN	1	20	multiple	0.58	0.02	135.54
CM038Q06	RNN	2	10	multiple	0.57	0.07	95.41
CM038Q06	RNN	2	20	multiple	0.51	0.01	137.34

Table S6. AUC, Kappa, and Training Time for LSTM Models with Multiple Features

Item	Model	Layer	Node	Input	AUC	Kappa	Time
CM015Q01	LSTM	1	10	multiple	0.89	0.61	5335.93
CM015Q01	LSTM	1	20	multiple	0.66	0.55	289.86
CM015Q01	LSTM	2	10	multiple	0.91	0.60	222.31
CM015Q01	LSTM	2	20	multiple	0.87	0.31	178.17
CM015Q02	LSTM	1	10	multiple	0.78	0.42	260.05
CM015Q02	LSTM	1	20	multiple	0.75	0.49	177.70
CM015Q02	LSTM	2	10	multiple	0.70	0.43	218.43
CM015Q02	LSTM	2	20	multiple	0.78	0.45	231.70
CM015Q03	LSTM	1	10	multiple	0.71	0.39	163.27
CM015Q03	LSTM	1	20	multiple	0.70	0.38	180.71
CM015Q03	LSTM	2	10	multiple	0.70	0.36	249.98
CM015Q03	LSTM	2	20	multiple	0.77	0.40	154.03
CM020Q01	LSTM	1	10	multiple	0.66	0.16	327.07
CM020Q01	LSTM	1	20	multiple	0.59	0.14	258.10
CM020Q01	LSTM	2	10	multiple	0.70	0.11	259.17
CM020Q01	LSTM	2	20	multiple	0.69	0.14	273.10
CM020Q02	LSTM	1	10	multiple	0.84	0.63	367.99
CM020Q02	LSTM	1	20	multiple	0.77	0.51	280.62
CM020Q02	LSTM	2	10	multiple	0.88	0.70	306.45
CM020Q02	LSTM	2	20	multiple	0.86	0.70	309.76
CM020Q03	LSTM	1	10	multiple	0.83	0.47	363.86
CM020Q03	LSTM	1	20	multiple	0.78	0.48	159.90
CM020Q03	LSTM	2	10	multiple	0.81	0.46	251.42
CM020Q03	LSTM	2	20	multiple	0.74	0.52	228.08
CM020Q04	LSTM	1	10	multiple	0.83	0.68	384.11
CM020Q04	LSTM	1	20	multiple	0.71	0.43	225.94
CM020Q04	LSTM	2	10	multiple	0.87	0.85	351.81
CM020Q04	LSTM	2	20	multiple	0.88	0.75	270.18
CM038Q03	LSTM	1	10	multiple	0.66	0.24	262.39
CM038Q03	LSTM	1	20	multiple	0.75	0.31	217.41
CM038Q03	LSTM	2	10	multiple	0.76	0.37	311.06
CM038Q03	LSTM	2	20	multiple	0.69	0.32	295.75
CM038Q05	LSTM	1	10	multiple	0.72	0.23	139.36
CM038Q05	LSTM	1	20	multiple	0.64	0.21	154.88
CM038Q05	LSTM	2	10	multiple	0.60	0.11	165.12
CM038Q05	LSTM	2	20	multiple	0.67	0.14	146.79
CM038Q06	LSTM	1	10	multiple	0.61	0.03	150.64
CM038Q06	LSTM	1	20	multiple	0.55	0.02	152.17
CM038Q06	LSTM	2	10	multiple	0.59	0.08	207.62
CM038Q06	LSTM	2	20	multiple	0.52	0.03	131.05

Table S7. AUC, Kappa, and Training Time for GRU Models with Multiple Features

Item	Model	Layer	Node	Input	AUC	Kappa	Time
CM015Q01	GRU	1	10	multiple	0.86	0.62	390.38
CM015Q01	GRU	1	20	multiple	0.84	0.55	241.60
CM015Q01	GRU	2	10	multiple	0.82	0.50	241.17
CM015Q01	GRU	2	20	multiple	0.75	0.53	201.25
CM015Q02	GRU	1	10	multiple	0.70	0.53	209.53
CM015Q02	GRU	1	20	multiple	0.80	0.51	167.37
CM015Q02	GRU	2	10	multiple	0.81	0.50	247.00
CM015Q02	GRU	2	20	multiple	0.80	0.43	289.04
CM015Q03	GRU	1	10	multiple	0.86	0.39	163.20
CM015Q03	GRU	1	20	multiple	0.77	0.36	104.79
CM015Q03	GRU	2	10	multiple	0.70	0.39	223.41
CM015Q03	GRU	2	20	multiple	0.79	0.38	221.03
CM020Q01	GRU	1	10	multiple	0.73	0.19	388.05
CM020Q01	GRU	1	20	multiple	0.67	0.13	264.32
CM020Q01	GRU	2	10	multiple	0.74	0.21	292.06
CM020Q01	GRU	2	20	multiple	0.74	0.12	256.39
CM020Q02	GRU	1	10	multiple	0.94	0.60	397.68
CM020Q02	GRU	1	20	multiple	0.80	0.53	297.04
CM020Q02	GRU	2	10	multiple	0.88	0.68	287.05
CM020Q02	GRU	2	20	multiple	0.88	0.72	275.68
CM020Q03	GRU	1	10	multiple	0.75	0.55	274.53
CM020Q03	GRU	1	20	multiple	0.83	0.47	195.60
CM020Q03	GRU	2	10	multiple	0.91	0.48	227.79
CM020Q03	GRU	2	20	multiple	0.84	0.53	253.49
CM020Q04	GRU	1	10	multiple	0.72	0.51	322.66
CM020Q04	GRU	1	20	multiple	0.77	0.45	210.20
CM020Q04	GRU	2	10	multiple	0.89	0.85	335.83
CM020Q04	GRU	2	20	multiple	0.82	0.84	307.76
CM038Q03	GRU	1	10	multiple	0.69	0.22	273.09
CM038Q03	GRU	1	20	multiple	0.54	0.22	215.34
CM038Q03	GRU	2	10	multiple	0.65	0.38	286.26
CM038Q03	GRU	2	20	multiple	0.73	0.34	200.73
CM038Q05	GRU	1	10	multiple	0.67	0.18	92.75
CM038Q05	GRU	1	20	multiple	0.68	0.12	85.19
CM038Q05	GRU	2	10	multiple	0.67	0.15	178.81
CM038Q05	GRU	2	20	multiple	0.69	0.19	176.42
CM038Q06	GRU	1	10	multiple	0.51	0.02	91.04
CM038Q06	GRU	1	20	multiple	0.58	0.05	100.81
CM038Q06	GRU	2	10	multiple	0.56	0.01	227.79
CM038Q06	GRU	2	20	multiple	0.59	0.01	186.84
